# Mechanistic Insights into the Photoisomerization of *N,N′*‐Disubstituted Indigos

**DOI:** 10.1002/chem.202200496

**Published:** 2022-03-29

**Authors:** Šimon Budzák, Justina Jovaišaitė, Chung‐Yang Huang, Paulius Baronas, Kamilė Tulaitė, Saulius Juršėnas, Denis Jacquemin, Stefan Hecht

**Affiliations:** ^1^ Department of Chemistry Faculty of Natural Sciences Matej Bel University Tajovkého 40 97401 Banska Bystrica Slovakia; ^2^ Institute of Photonics and Nanotechnology Vilnius University Saulėtekis av. 3 LT-10257 Vilnius Lithuania; ^3^ Institute for Chemical Reaction Design and Discovery (WPI-ICReDD) Hokkaido University Kita 21, Nishi 10, Kita-ku Sapporo Hokkaido 001-0021 Japan; ^4^ CEISAM Lab, UMR 6230 Université de Nantes, CNRS 44000 Nantes France; ^5^ Department of Chemistry & IRIS Adlershof Humboldt-Universität zu Berlin Brook-Taylor-Strasse 2 12489 Berlin Germany; ^6^ DWI – Leibniz Institute for Interactive Materials Forckenbeckstrasse 50 52074 Aachen Germany; ^7^ Institute for Technical and Macromolecular Chemistry RWTH Aachen University Worringer Weg 2 52074 Aachen Germany

**Keywords:** computational chemistry, dyes/pigments, indigo, photochromism, photophysics

## Abstract

*N,N′*‐disubstituted indigos are photoswitchable molecules that have recently caught the attention due to their addressability by red‐light. When alkyl and aryl groups are utilized as the *N*‐substituents, the thermal half‐lives of *Z* isomers can be tuned independently while maintaining the advantageous red‐shifted absorption spectra. To utilize these molecules in real‐world applications, it is of immense importance to understand how their molecular structures as well as the environment influence their switching properties. To this end, we probed their photoisomerization mechanism by carrying out photophysical and computational studies in solvents of different polarities. The fluorescence and transient absorption experiments suggest for more polar excited and transition states, which explains the bathochromic shifts of absorption spectra and shorter thermal half‐lives. On the other hand, the quantum chemical calculations reveal that in contrast to *N*‐carbonyl groups, *N*‐alkyl and *N*‐aryl substituents are not strongly conjugated with the indigo chromophore and can thus serve as a tool for tuning the thermal stability of *Z* isomers. Both approaches are combined to provide in‐depth understandings of how indigos undergo photoswitching as well as how they are influenced by *N*‐substituent and the chemical surroundings. These mechanistic insights will serve as guiding principles for designing molecules eyeing broader applications.

## Graphical abstract

**Indigo photoswitches** can undergo photoisomerization triggered by red‐light, which is of immense potential in applications such as biomedical research and material science. In this mechanistic investigation, the influence of chemical environment and *N*‐substituents is elucidated through photophysical and computational approaches. This study further provides insights into the course of photoisomerization.

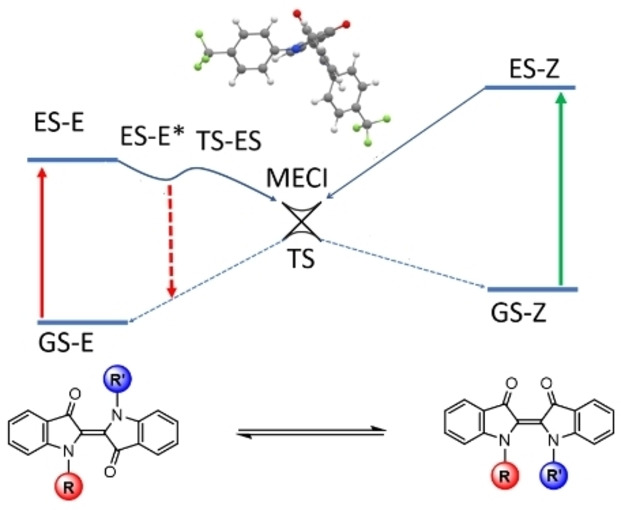

## Introduction

**Scheme 1:**
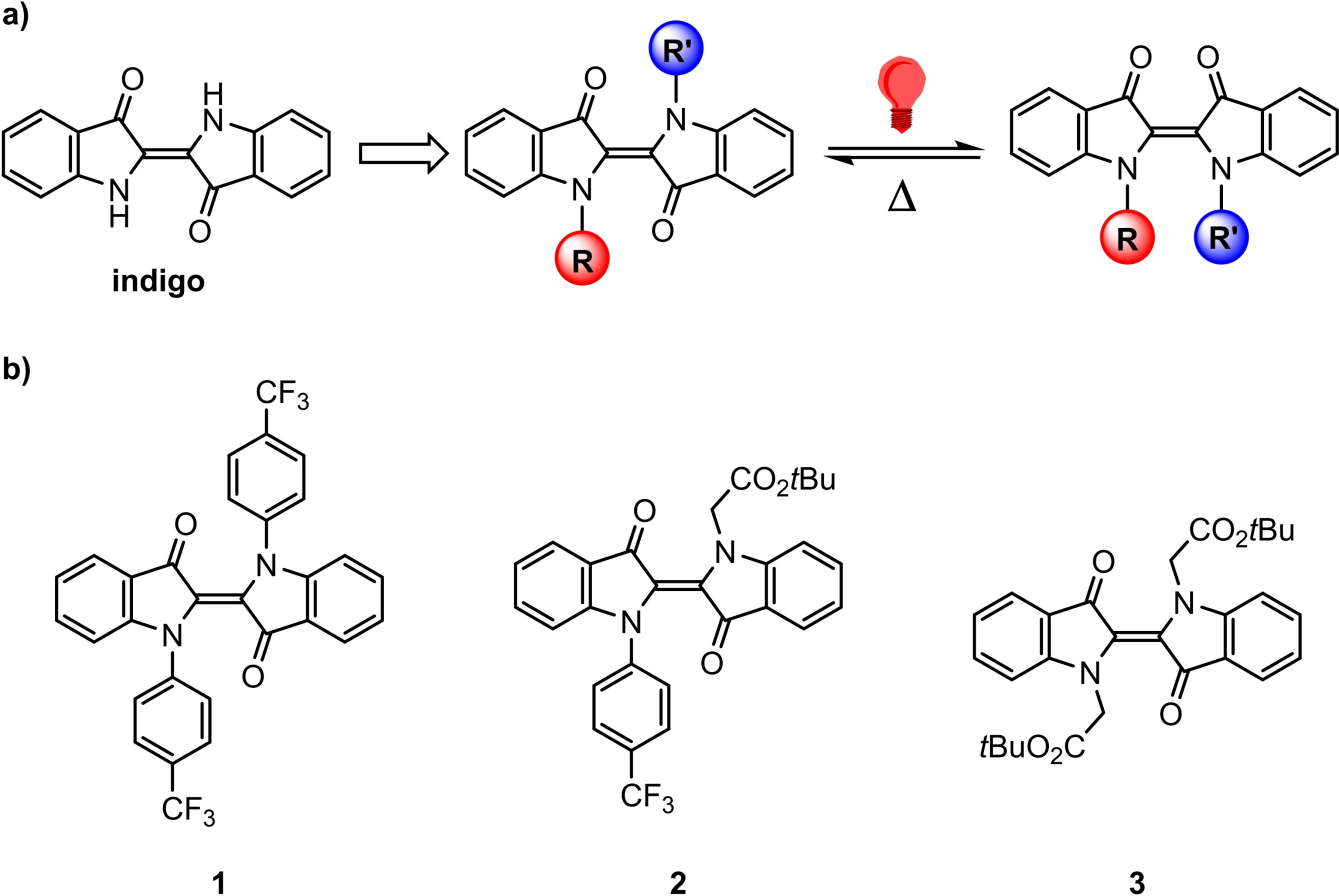
a) Indigo photoswitches and b) *N,N′*‐disubstituted indigos 1–3 investigated in this study.

Indigo, one of the most ancient dyes developed in human history, possesses unique functional and chemical features. It was found that when both N−H hydrogen atoms are substituted, these derivatives undergo *E*–*Z* photoisomerization upon illumination with red‐light (Scheme 1).^[1]^ In some recently reported mono‐*N*‐arylated indigos, *E*–*Z* photoisomerization is able to successfully compete with excited state proton transfer of the unfunctionalized N−H moiety.^[2]^ The fact that these photochromic indigo derivatives can be switched by long‐wavelength irradiation is of immense value for biomedical^[3]^ and material^[4]^ applications. The lower excitation energy minimizes radiation damage on the one hand and allows for enhanced penetration depth due to their negative photochromism on the other hand.^[5]^ Moreover, there are dramatic structural differences between the interconverting *E* and *Z* isomers of such indigo photoswitches that can be exploited. We underline that structurally related hemiindigos have recently been developed as photoswitches by the Dube group.^[6]^

In the literature, indigo photoswitches include *N,N′*‐dialkyl,^[7]^ *N,N′*‐diacyl,^[8]^ and *N,N′*‐di‐(*tert*‐butyloxycarbonyl) (BOC)^[9]^ compounds as well as the special cases of *N*‐aryl‐*N*−H indigos^[2]^ and *N*‐alkyl‐*N*−H indigos.^[10]^ The *N,N′*‐dimethylindigo gives a *Z* isomer that is extremely thermally unstable. In contrast, both *N,N′*‐diacyl and *N,N′*‐di‐BOC‐substitutions result in thermally long‐lived *Z* isomers, presumably due to the resonance stabilization between nitrogen lone pairs and the carbonyl groups. However, the consequence of these prolonged thermal half‐lives are the accompanying blue‐shifted absorption spectra, meaning that these indigos can no longer be switched using red‐light. To address this issue, some of us developed a series of indigo compounds substituted with *N*‐alkyl and *N*‐aryl groups and demonstrated the decoupling of substituent effects on absorption spectra and thermal half‐lives.^[11]^ Thus, the thermal stability of the *Z* isomers can be controlled while the parent *E* isomers remain switchable by red‐light. In particular, we discovered that the installment of electron‐withdrawing groups on the *N*‐aryl substituents could extend the thermal half‐lives, which provides a strategy to rationally design indigo photoswitches. A preliminary study revealed the uniqueness of these *N*‐substituents resides in their ability to modulate the indigo chromophore through inductive rather than resonance effects.

In order to further rationalize the working principles of indigo photoswitches, it is imperative to fully understand the mechanism by which they isomerize. Specifically, we wish to decipher how these *N*‐alkyl and *N*‐aryl substituents influence the switching behavior. In addition, our studies should provide insight into how the environment of these indigos influences their switching properties – a question important to answer when targeting material and biological applications. In this context, we want to highlight a recent study conducted on *N,N′*‐diacylindigos and *N,N′*‐dibenzoylindigos, where the authors showed significant involvement of *N*‐carbonyl groups in the switching mechanism of these blue‐shifted compounds.^[12]^ An investigation on the red‐shifted compounds, which are arguably more advantageous, should therefore provide useful information for the further development of this class of photoswitchable molecules. Herein, we present a comprehensive experimental and theoretical investigation on *N,N′*‐disubstituted indigos carrying *N*‐alkyl as well as *N*‐aryl substituents.

## Results and Discussion

### Photoisomerization behavior

Three representative indigo photoswitches covering the combinations of *N*‐aryl and *N*‐alkyl substituents were chosen for this study (compounds 1, 2, and 3 in Scheme 1). First, their switching behavior was studied in four different solvents (Table 1; see Section II in the Supporting Information for more details). These *N*‐alkyl and *N*‐aryl indigos switch efficiently in both polar and non‐polar solvents. The absorption spectra of the *E* isomers of all three compounds undergo bathochromic shifts (Δ*λ*_max_) as the solvent polarity increases, with the *Z* isomers undergoing larger shifts than their *E* configured counterparts. Moreover, the thermal half‐lives decrease significantly in the more polar solvents; for example, for compound 2, the thermal half‐life dropped from 9.9 min in toluene to 1.7 min in DMSO. As a result, the investigated dyes show higher photoconversion, i. e., larger *Z* content in the photostationary state (PSS), in non‐polar solvents. This finding suggests that the excited state structures involved in the photoisomerization processes (E* and Z*) as well as the transition state structure corresponding to the thermal process (TS^≠^) are more polar than the ground state minima (*E* and *Z*). Furthermore, by conducting a linear free energy analysis where the thermal half‐lives of the three compounds were measured at four different temperatures in MeCN, we were able to determine the activation energies for the thermal‐back reaction (Table S1).

**Table 1:** Thermal half‐lives, dark‐ and PSS−Z%, and *λ*_max_ of indigo photoswitches 1–3 in different solvents.

Compound	Solvent^[a]^	*t*_1/2_ [min]^[b]^	Dark *Z*%	PSS Z%	*λ*_max_‐*E* [nm]	*λ*_max_‐*Z* [nm]	Δ*λ*_max_ [nm]	*ϵ_E_* [M^−1^ cm^−1^]
1	Toluene	151	14 %	80 %	620	565	55	9500
	CH_2_Cl_2_	78	32 %	77 %	626	577	49	7500
	MeCN^[c]^	81	27 %	83 %	628	589	39	12900
	DMSO	18	21 %	73 %	635	580	50	9600
2	Toluene	9.9	0 %	81 %	622	551	71	14500
	CH_2_Cl_2_	5.7	0 %	70 %	628	569	59	13800
	MeCN^[c]^	3.5	0 %	56 %	622	572	50	13200
	DMSO	1.7	0 %	58 %	632	580	52	13500
3	Toluene	4.1	0 %	91 %	621	540	81	16300
	CH_2_Cl_2_	3.4	0 %	86 %	626	560	66	15800
	MeCN^[c]^	2.8	0 %	77 %	623	561	62	15700
	DMSO	1.1	0 %	76 %	634	573	61	14800

[a] Dielectric constants for: toluene: *ϵ*=2.38, CH_2_Cl_2_: *ϵ*=8.93, MeCN: *ϵ*=37.5, DMSO: *ϵ*=46.7. [b] *t*(eq)_1/2_ for compound 1. [c] Data acquired in MeCN are from Ref. [11].

### Photophysical evaluation

**Figure 1:**
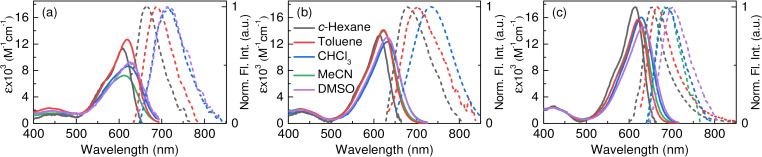
Absorption (solid lines) and normalized fluorescence spectra (dashed lines) of indigo photoswitches a) 1, b) 2, and c) 3 in solvents of different dielectric constant. Absorption spectra correspond to the *E*/*Z* mixture for indigo 1 and to pure *E* isomer for indigos 2 and 3. For the measurements of fluorescence spectra, samples in *c*‐hexane, toluene and MeCN were excited at 620 nm, while samples in CHCl_3_ and DMSO were excited at 630 nm. Fluorescence spectra of 1 in MeCN and 2 in MeCN and DMSO are not depicted as the emission intensity was too low to record these spectra. The legend indicated in b) is also applicable for a) and c).

Steady‐state absorption and fluorescence spectroscopy experiments were performed in five different solvents (Figure 1 and Table 2). In accord to Table 1, in all solvents, compound 1 exists as an *E*/*Z* mixture, whereas 2 and 3 are purely *E*‐configured. Absorption spectra of all three compounds are located in a similar spectral range, maintaining an advantageous redshift, as already described in our previous work.^[11]^ All absorption spectra show a slight bathochromic shift upon increasing the dielectric constant of the solvent. The absence of *N*‐aryl groups (compound 3) leads to the largest oscillator strength, as clearly seen from an increased molar extinction coefficient.

**Table 2:** Steady‐state spectroscopy data of indigo photoswitches 1–3 in various solvents and in 1 wt % Zeonex.

Compound	Solvent^[a]^	*λ*_abs, max_ [nm]	*λ*_fl, max_ [nm]^[b]^	*Φ*_fl_ [%]^[c]^
1	*c*‐Hexane	610	665	0.1
	Toluene	620	687	0.2
	CHCl_3_	622	714	0.1
	MeCN	612	–	–
	DMSO	626	717	0.1
	1 wt % Zeonex	611	663	5
2	*c*‐Hexane	615	674	0.6
	Toluene	622	704	0.2
	CHCl_3_	631	736	0.1
	MeCN	622	–	–
	DMSO	632	–	–
	1 wt % Zeonex	616	670	4
3	*c*‐Hexane	615	651	9.1
	Toluene	621	666	3.2
	CHCl_3_	628	687	0.5
	MeCN	623	682	0.2
	DMSO	634	702	0.2
	1 wt % Zeonex	618	657	11

[a] Dielectric constants for: *c*‐Hexane: *ϵ*=2.02, toluene: *ϵ*=2.38, CHCl_3_: *ϵ*=4.81, MeCN: *ϵ*=37.5, DMSO: *ϵ*=46.7. [b] Fluorescence measurements of certain compounds are not available due to low emission intensity in these solvents.

Pronounced differences of the excited state dynamics are observed in the fluorescence spectra and fluorescence quantum yields. The fluorescence spectra are red‐shifted by ca. 50–60 nm between non‐polar *c*‐hexane and polar DMSO, indicating that the excited indigo dyes are more polar than in the ground state. The most redshifted fluorescence spectra are attributed to indigo 2 with only one *N*‐aryl substituent, while the absence of *N*‐aryl moieties in 3 shifts the emission spectra to shorter wavelengths.

Indigo dyes 1 and 2 show only negligible fluorescence quantum yields from 0.1 % to 1 % in all tested solvents, characteristic to a parent indigo in the keto (neutral) form and its *N,N′*‐disubstituted derivatives.[^12^, ^13^] However, an unusually high fluorescence quantum yield of 9.1 % with regard to indigo dyes was observed for 3 in *c*‐hexane, which gradually decreases along with an increasing dielectric constant of the surrounding medium, down to ca. 0.2 % in MeCN and DMSO (as observed for *N,N′*‐Boc‐indigos^[9]^). The highest fluorescence quantum yield of compound 3 is potentially related to the absence of aryl moieties that can undergo rotation and serve as an additional non‐radiative decay channel.^[14]^

The physical incorporation of indigo dyes in a rigid polymer matrix (1 wt % Zeonex, see the General methods in the Supporting Information) allowed us to freeze the dye molecules in their ground state geometry, thereby strongly limiting their ability to move. The obtained steady‐state absorption and fluorescence spectra (Table 2 and Figure S1) are almost identical to those of 1, 2, and 3 in c‐hexane. However, for indigos 1 and 2, the fluorescence quantum yields are 10–15 times higher in the polymer matrix as compared to the non‐polar solvent, most probably due to restricted vibrations of the aryl moieties, whereas for the non‐arylated compound 3 the fluorescence quantum yield resembles the one found in *c*‐hexane. This results clearly demonstrates the crucial role of the *N*‐substituents and their interaction with the chemical surrounding for the excited state dynamics of indigos.

**Figure 2:**
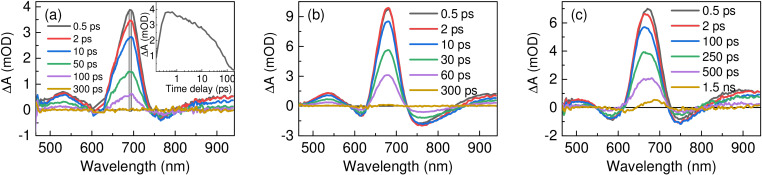
Transient absorption spectra at different time delays of indigo photoswitches a) 1, b) 2, and c) 3 in toluene. Inset in a) shows transient signal recorded at 693 nm. Laser excitation wavelength was set to 620 nm.

Deeper insights into the excited state deactivation mechanism of indigo photoswitches could be gathered by employing transient absorption (TA) experiments. The TA spectra at different time delays are presented exemplarily for toluene in Figure 2 and for other solvents in the Supporting Information (Figure S2). The lifetimes of TA signals were extracted by employing global analysis using a sequential model (Figure S3)^[15]^ with two decay components *τ*_1_ and *τ*_2_(E*) (Table 3). The obtained TA spectra for all three compounds in toluene, CHCl_3_, MeCN, and DMSO are similar to each other and do not show any significant spectral shape changes depending on substituent or solvent parameters. The stimulated emission (SE) for indigo molecules is detected at 720–850 nm. The excited state absorption (ESA), attributed to singlet transitions occurring in the E* isomer,[^1b^, ^16^] is present at 650–700 nm (as well as 500–590 nm), similar to what has already been observed for *N,N′*‐diacetylindigo[^12^, ^17^] and *N,N′*‐dibenzoylindigo.^[12]^

**Table 3:** Transient absorption signal decay constants of indigo photoswitches 1–3 in different solvents.

Compound	Solvent	*τ*_1_ [ps]	*τ*_2_ (E*) [ps]
1	Toluene	3.5	61
	CHCl_3_	2.6	37.0
	MeCN	1.3	18.8
	DMSO	1.9	39.1
2	Toluene	0.6	50.7
	CHCl_3_	1.5	22.5
	MeCN	1.7	9.2
	DMSO	1.7	20.9
3	Toluene	2.0	448.3
	CHCl_3_	2.0	98.2
	MeCN	0.7	40.6
	DMSO	1.3	60.6

The inset of Figure 2(a) shows that the ESA signal consists of fast and slow decay components, and this holds for the SE signal. The fast initial decay component *τ*_1_ is in the range of 0.6–3.5 ps (Table 3), which is in a comparable timescale as solvation dynamics^[18]^ and might be related to solvent effects and/or structural relaxation. The slower component *τ*_2_(E*) corresponds to the lifetime of the excited *E* isomer (E*) and depends on both the substitution pattern and the solvent polarity. Two *N*‐alkyl groups lead to the longest lifetimes of E* (for compound 3, *τ*_2_(E*)=448.3 ps in toluene), while the presence of one or two *N*‐aryl substituents results in significantly shorter lifetimes (for 1 and 2, *τ*_2_(E*)=61 ps and 51 ps, respectively, in toluene) and thus a more efficient non‐radiative decay. Furthermore, the E* lifetimes of all compounds are very sensitive to the polarity of the medium: the higher the dielectric constant, the lower *τ*_2_(E*). For example, going from toluene to MeCN, *τ*_2_(E*) drops by a factor of three for indigo 1, a factor of five for indigo 2, and a factor of ten for indigo 3. As it will be shown later, the barrier for twisting around C=C bond is reduced in more polar solvents, which is consistent with shorter lifetimes for E*. Note that the results in the rather polar DMSO do not follow the same trend, presumably due to its high viscosity.

The TA measurements of samples in a rigid environment of 1 wt % Zeonex showed prolonged E* lifetimes up to several nanoseconds. No fast component was present, confirming that *τ_1_* observed in solvents is related to molecular reorganization and solvent effects (Figure S4). The obtained fluorescence decay profiles for thin film samples are biexponential for 1 and 2, displaying a variety of frozen conformers (Figure S5). On the other hand, the rigid sample of 3 demonstrates the highest uniformity of molecules, as the fluorescence decay profiles can be fitted mono‐exponentially with a time constant of 4.8 ns. The increased fluorescence quantum yields along with increased fluorescence lifetimes suggest for the restricted non‐radiative decay channel in the rigidified environment of the polymer matrix.

The photophysical study of indigo dyes in solvents revealed the great possibilities to control excitation deactivation by employing either different *N,N′*‐substituents or by changing the polarity of environment. The *N*‐alkyl groups yield the highest fluorescence quantum yields and the longest E* lifetimes. In contrast, *N*‐aryls substituents bathochromically shift fluorescence spectra and boost the efficiency of non‐radiative deactivation. The increased polarity of solvents also causes a slight redshift of fluorescence spectra, accompanied by a drop of fluorescence quantum yields and reduced lifetimes of E*. Furthermore, the optical properties of indigo dyes can be well controlled by the rigidity of surrounding medium as both the fluorescence quantum yields and the fluorescence lifetimes are increased. Nevertheless, the rather low efficiency of the fluorescence, even in a rigidified polymer matrix, points to the existence of an additional and efficient non‐radiative deactivation pathway, for example, intersystem crossing to the corresponding triplet excited states.

### Quantum chemical calculations

To gain further insight into the photophysical properties of the investigated indigos, we resorted to computational methods (for details, see Computational methods and additional methods in the Supporting Information). First, the thermal *Z*→*E* process was simulated. The calculated thermal *Z*→*E* activation energies are listed in Table 4. The highest barrier is obtained for indigo 1, followed by indigos 3 and next 2, which is in line with experimental determination of the activation energies in acetonitrile.

**Table 4:** Computed activation energies and experimental thermal half‐lives for thermal *Z*→*E* isomerization.

Compound	1		2		3	
	*Ε*_a_ [kcal/mol]	*t*(eq)_1/2_ [min]	*Ε*_a_ [kcal/mol]	*t*_1/2_ [min]	*Ε*_a_ [kcal/mol]	*t*_1/2_ [min]
Toluene	24.6	151	18.7	9.9	21.3	4.1
CH_2_Cl_2_	24.5	78	19.3	5.7	20.5	3.4
MeCN^[a]^	24.4 (24.3)	81	19.4 (19.3)	3.5	20.1 (22.0)	2.8
DMSO	24.3	18	19.4	1.7	20.1	1.1

[a] Experimentally determined values from linear free energy analysis in MeCN are shown in parathesis. See Supporting Information for more details.

**Figure 3:**
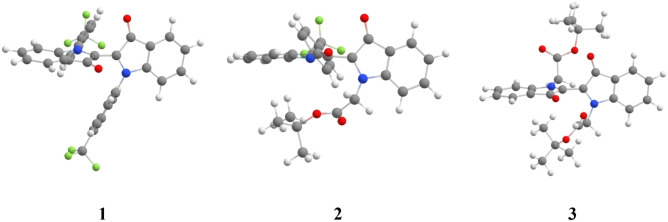
BS‐DFT thermal transition state structure for studied molecules.

As expected, the transition state structure features two almost perpendicular “semi‐indigo” moieties (Figure 3). The calculated magnitude of the dipole moment of 3 is larger for the transition state structure (11.0 D in acetonitrile) than for the *Z* isomer (5.8 D in acetonitrile, Table S3), explaining decrease of half‐life in more polar solvents. Moreover, the polar oxygen atoms from the t‐butyl ester group are less solvent accessible in the *Z* isomer structure, which likely contributes to the smaller stabilization in polar media. A similar trend is observed for 1 although the difference in dipole moments is smaller. For compound 2 theory predicts an opposite trend as compared to experiment. However, the observed changes of half‐lives correspond to a change in activation Δ*G* smaller than 1.5 kcal/mol, which is of the order of accuracy of PCM‐DFT. Theoretical estimates of the solvent influence on the activation energies show smaller than experimental decrease in 1, alike decrease for 3, and as mentioned before, opposite behavior compared for compound 2. Increased basis set size, selection of a different solvent model and other theoretical methods were tested but without significant improvement, suggesting that the overall agreement with activation energies does not apply to the fine differences between solvents, a likely consequence of the inherent limits of continuum models.

**Figure 4:**
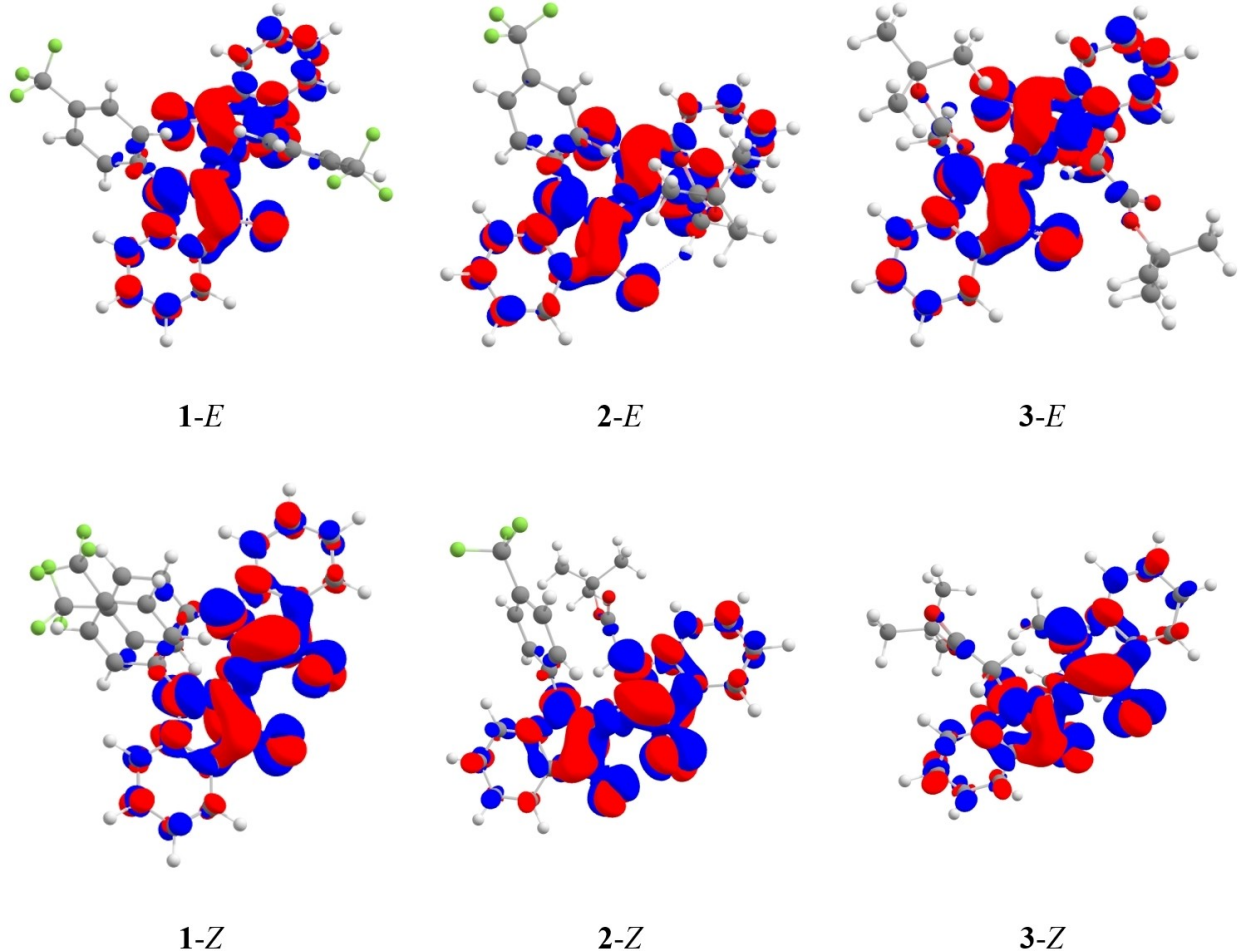
Electron density differences for the first excited state of studied molecules. Red/blue colors show increase/decrease of electron density. A contour value of 0.0012 was applied. See the Supporting Information for orbital representations.

The ωB97X‐D vertical excitation energies to the lowest excited states of both isomers were calculated, and as expected, smaller excitation energies are found for the *E* isomer (Table S4). The theoretical wavelengths are too small as compared to experiment, a logical consequence of neglecting vibronic couplings in the theoretical protocol. More importantly, in all cases, theory predicts bathochromic shifts of the excitation energies when the solvent polarity increases, which relates to the higher polarity of the excited state as compared to the ground state. This trend is consistent with the experimental results (Table 2). The electron density difference plots (Figure 4) reveal a dominant π→π* transition that is almost exclusively located on the core of the indigo structure, in line with the well‐known H‐shaped chromophore of indigo derivatives.^[18]^ The phenyl rings as well as the ester groups therefore tune the excitation mostly through inductive effects. We do not observe any significant charge‐transfer character in these transitions. In all cases, only a small fraction of electron shifts (Table S5), leading to a charge‐transfer distance of ca. 1 Å only. Comparing the present molecules to *N,N′*‐di‐(tert‐butyloxycarbonyl)indigo (BOC‐indigo): In the BOC‐indigo, one can observe a larger involvement of the *N*‐carbonyl in the excitation, contrasting the present dyes in which the π electrons of the *N*‐substituent are decoupled from the indigo core (Figures 4, S6 and S7). This observation again shows the uniqueness of *N*‐aryl and *N*‐alkyl substituents as compared to *N*‐carbonyl‐type groups: by modulating the π system through inductive rather than resonance effects, they successfully allow tuning thermal half‐lives of indigo photoswitches without sacrificing their redshifted character.

**Figure 5:**
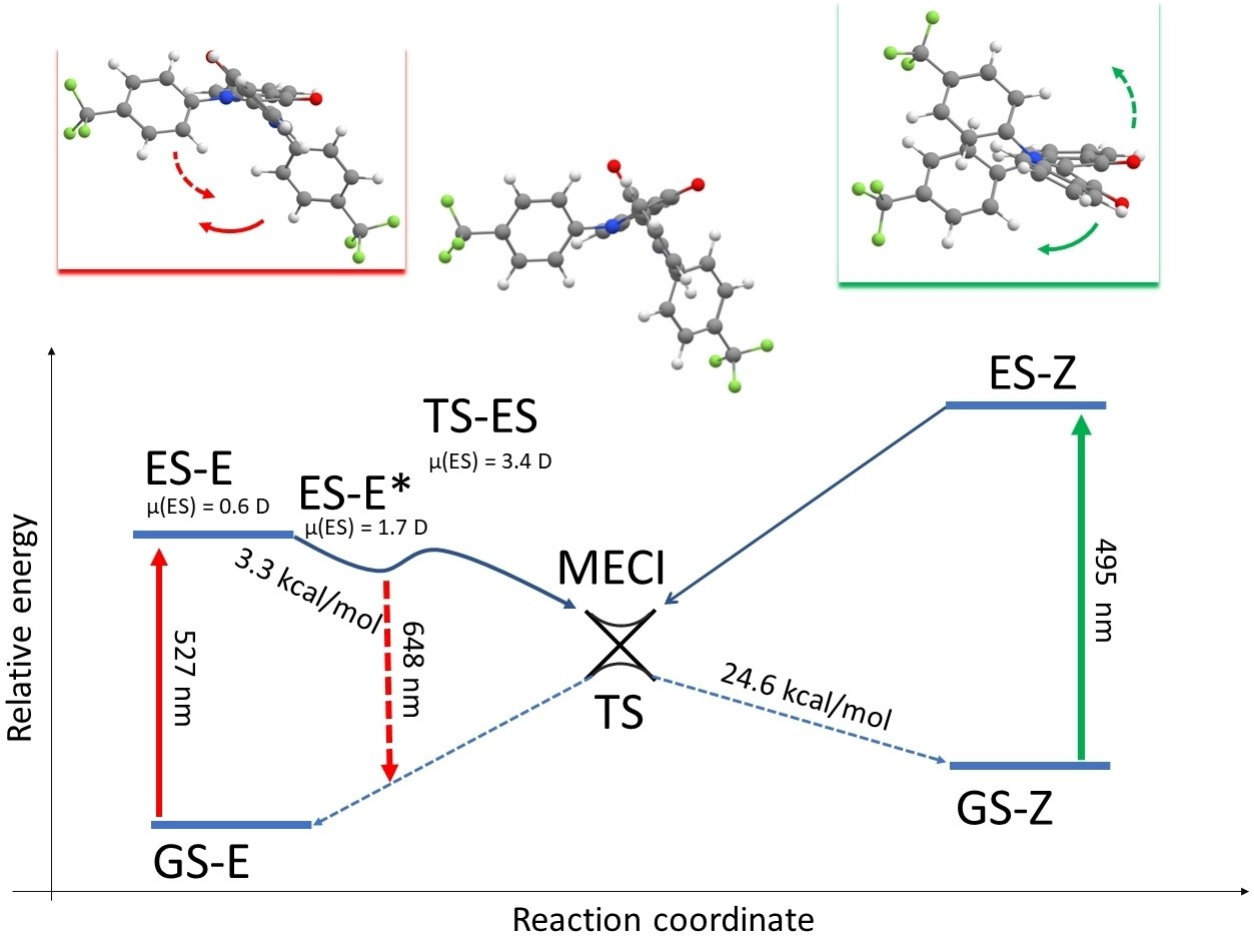
Schematic depiction of the ground and excited state arrangements for the compound 1 in toluene. Insets show *E* and *Z* isomers of molecule 1, arrows point to the rotation towards MECI. The values of the excited state dipole moment for this molecule are shown together with calculated activation energy for thermal *Z*→*E* isomerization and vertical excitation/emission wavelengths.

In the ground state, the *E* isomer is the most stable of all molecules. The excitation to the first excited state transfers electron density to the π* antibonding orbital and decreases the bond order of central C=C bond from double to single. Such a scenario usually leads to fast non‐radiative deactivation of the excited state through twisting. Nevertheless, a stable though shallow minimum in the excited state could be located for all *E* isomers in the calculations which is consistent with the observation of fluorescence for all *E* isomers experimentally. The wavelengths of the fluorescence emission are in good agreement between theory and experiment (Table 5). Following the rotation around the central C=C bond, each molecule either: i) pushes two bulky substituents against each other when following the minimal energy path, or ii) passes through a conformation where the C=O and N−R moieties directly face each other (see the animation file in the Supporting Information). Both possible rotational directions are therefore protected by a barrier, and a stable population of the *E* isomer might indeed exist for a non‐trifling time. Initial gradients from the Franck‐Condon region, however, move all molecules in the direction of (i). This analysis is in line with the experimental observation of a relatively long lifetime for ester‐substituted indigo 3 (445 ps) and much shorter lifetime for indigo 1 (75 ps). As an illustration, the central C−C=C−C dihedral angle of indigo 3 decreases from 167° in the ground state to 136° in the excited state. The conical intersection is located at a dihedral angle of 93°, though we stress that this minimum energy conical intersection (MECI) also includes pyramidalization of one carbon atom of the C=C central bond. The redshift of the emission is not only due to higher polarity of the excited state but also to the relaxation of the excited state geometry. Indeed, we note that for indigo 3, the excited state dihedral value decreases from 136° in toluene to 130° in DMSO (see Table S6). In more polar solvents, the indigo photoswitches explore regions relatively close to the photochemical funnel, which logically correlates with shorter excited state lifetime and decreases of the fluorescence quantum yield. The ground state energy surface that can be reached through the MECI (Figure 5) gives access to both *E* and *Z* isomers. The MECI is protected by a small barrier, and the structure of the excited state transition state (ES‐TS) is more polar as compared to the corresponding excited state *E* structure, thus the more polar solvent selectively stabilizes this transition state and further decreases the barrier in the excited state.

**Table 5:** Calculated vertical emission wavelengths of the studied molecules, the experimental *λ*_fl_ are given in parentheses. All values in nm.

Solvent/Compound	1		2	3
	*E*	*Z*	*E*	*E*
Toluene	648 (687)	–	643 (704)	586 (666)
CH_2_Cl_2_	668	–	661	601
MeCN	679	741	665	609 (682)
DMSO	680 (717)	742	665	610 (702)

When comparing the emission maxima, one notes that indigo 3 presents the most blue‐shifted spectrum, which may be related to the bulky substituents limiting the possibility of relaxation through rotation around the central bond. Indeed, this dihedral angle attains 154° in the excited state, i. e., is slightly larger than in indigo 1 (150°) which presents a comparatively redshifted emission despite both having vertical excitations very close from one another at the Franck‐Condon point. Theory predicts indigo 1 to be the most redshifted emitter, mostly because of the large relaxation in the excited state. Experimentally, however, indigo 2 emits at the longest wavelength of all considered dyes. The theoretical analysis is complicated here due to the various possible local minima corresponding to different rotations of the *N*‐substituents. For example, two excited state local minima of indigo 2 (Figure S8) differing merely by 0.4 kcal/mol are easily accessible. These two minima show central dihedral angle differing by more than 5°, and fluorescence emission wavelengths differing by 30 nm. This difference is observed since the excited state potential energy surface is very flat while the ground state energy changes considerably when modifying the dihedral angle.

A different picture is found after the excitation of the *Z* isomer. While the *Z* isomer is energetically less stable than its *E* counterpart in the excited state (as in the ground state), there is no double bond preventing its rotation (in contrast to the ground state), so that geometrical relaxation is easy. The (spin‐flip‐TD‐DFT) geometry optimizations of the *Z* excited state for both indigos 2 and 3 that we started from the Franck‐Condon region directly ended up in a conical intersection geometry. For indigo 1, we could locate an excited state minimum for the *Z* form in acetonitrile and DMSO. The corresponding emission energy has a large Stokes shift of more than 6600/cm, and interestingly, the minimum is stabilized thanks to dispersion interactions between the two phenyl rings only, thus likely it is very easily bypassed in the real‐life dynamics due to its shallowness.

In short, the theoretical calculations not only correlate nicely with the key experimental observations but also highlight that a very subtle combination of effects is responsible for the variations noticed between the three investigated systems.

## Conclusion

By modifying their molecular structure as well as their environment, the properties of photoswitches can often be rationally manipulated within a certain deviation from their default character. When developing a new photoswitchable system, it is thus imperative to understand how both, the chemical structure including substitution pattern and the surroundings, in particular the solvent, impart such an influence. In this manuscript, we summarize our comprehensive studies on *N,N′*‐disubstitued indigos that have recently been shown to display red‐light addressability and tunable thermal stability.^[11]^ Specifically, the combination of steady‐state and ultrafast time‐resolved spectroscopy with first‐principle calculations revealed the impact of the environment as well as of the *N*‐substituents on the switching behavior of indigos through controlling both the excited *E* isomer twisting barrier around C=C central bond and the energy of transition state. The polar solvents along with *N*‐aryl substituents grants an easier access to the key conical intersection once the *E* isomer is excited, consequently leading to the rapid non‐radiative deactivation. On the other hand, the *N*‐alkyl substituted indigo has to overcome a higher twisting barrier, yielding a stable E* population and thus fluorescence quantum yields and E* lifetimes at least ten times higher in a non‐polar as compared to a polar environment. Furthermore, the highest *Z→E* thermal barrier is accounted for *N*‐aryl substituted indigos, causing the longest thermal half‐lives of *Z* isomer. Importantly, the unique impact of the *N*‐alkyl and *N*‐aryl substituents can be related to their *indirect* participation to the chromophore: By adjusting the energy levels through inductive effect instead of directly coupling to the indigo core through resonance, these *N*‐substituents allow to finetune the thermal half‐lives of indigo photoswitches without losing their desirable red‐shifted absorption character. In addition, the energy of the transition state and thus the thermal half‐live of the *Z* isomer is decreased in more polar solvents. This renders indigos attractive candidates for biological applications that rely on activity only during red illumination but rapid deactivation in the dark, i. e., a succinct ON‐OFF switching with one wavelength only that moreover is harmless to cells and deeply penetrating into tissue. These key properties of indigos illustrate that this emerging class of photoswitches can be designed to respond to different chemical surroundings as experienced when exploiting them in various applications. We thus aim to take indigos to the next level by exploring the full potential of these red‐light photoswitches in biological settings and material science.

## Conflict of interest

The authors declare no conflict of interest.

1

## Supporting information

As a service to our authors and readers, this journal provides supporting information supplied by the authors. Such materials are peer reviewed and may be re‐organized for online delivery, but are not copy‐edited or typeset. Technical support issues arising from supporting information (other than missing files) should be addressed to the authors.

Supporting Information

## Data Availability

The data that support the findings of this study are available in the supplementary material of this article.
