# Supplementary material for: Mechanistic Insights into the Photoisomerization of N,N′‐Disubstituted Indigos
Source: Chemistry. 2022 Mar 29;28(26):e202200496. doi: 10.1002/chem.202200496 (PMC9311193; doi:10.1002/chem.202200496)
Supplement: Supplementary file 1 — Supporting Information [file CHEM-28-0-s001.pdf]

Chemistry–A European Journal

Supporting Information

Mechanistic Insights into the Photoisomerization of *N,N′*-Disubstituted Indigos

Šimon Budzák, Justina Jovaišaitė, Chung-Yang Huang, Paulius Baronas, Kamilė Tulaitė, Saulius Juršėnas,* Denis Jacquemin,* and Stefan Hecht*

Table of Contents

I. General Methods SI-2

II. UV-vis Spectroscopy in DMSO, CH_2Cl_2, and Toluene SI-4

III. Linear Free Energy Analysis SI-7

IV. Photophysical Data SI-11

V. Computational Methods and Additional Data SI-15

VI. References SI-21

VII. XYZ Coordinates SI-22

I. General methods

UV-vis spectroscopy

UV-vis spectroscopy was performed in quartz cuvettes (1.00 cm) on Agilent Cary 50 and Cary 60 instruments equipped with Peltier-thermostated cell holder (temperature accuracy ± 0.1 K). All the UV/Vis absorption spectra were recorded in the Cary 50 instrument, and a 660-nm mounted-LED (Thorlabs, M660L4, 660 nm, FWHM = 20 nm, 940 mW min, 1200 mA) in combination with a light cable was utilized as the irradiation source. The Cary 60 instrument was equipped with a waveguide (orthogonal attachment with respect to the measuring beam) connected to a LOT Monochromator MSH-300 with variable slit (set to 5 mm), and a LOT 500 W Hg high-pressure arc light source was used.

Sample preparation

Photophysical measurements were performed on solutions and thin films. Solvents used for photophysical measurements (cyclohexane, toluene, chloroform, dichloromethane, acetonitrile and dimethylsulfoxide) were purchased from Sigma-Aldrich and were used as received. Concentrations were selected according to performed experiment (as indicated below).

Abbreviations: *c*-hexane (cyclohexane), CH_2Cl_2 (dichloromethane), $CHCl_3$ (chloroform), MeCN (acetonitrile), DMSO (dimethylsulfoxide).

Thin films were drop casted from 1% wt. Zeonex and toluene solution on quartz plates and kept in the dark while drying.

Steady-State experiments

Absorption spectra were recorded using UV–vis–near IR spectrophotometer Lambda 950 (PerkinElmer). Fluorescence spectra were measured using a back-thinned CCD spectrometer PMA-11 (Hamamatsu). Samples were excited by using a Xe lamp (FWHM < 10 meV) coupled with monochromator. The concentration of $5x10^{-5}$ M was used for absorption and fluorescence spectra measurements.

Fluorescence quantum yields (FL QYs) of solutions were determined by comparative method, using Nile blue perchlorate (in acidic ethanol, QY=0.27[1]) as a reference material. Sample and reference concentrations for FL QYs experiments were selected so that the optical density (OD) would be in a range from 0.03 to 0.08.

The experiments were performed using 1 cm quarts cuvettes.

FL QYs of thin films were determined by integrated sphere method.

Time-resolved fluorescence experiments

Time-resolved fluorescence measurements in a nanosecond time domain were performed with Streak Scope C10627 detector (Hamamatsu) exciting samples with 580 nm femtosecond laser pulses (Light Conversion Pharos-SP and Orpheus).

Transient absorption spectroscopy

Femtosecond time-resolved differential absorption (TA) measurements were carried out using a Harpia pump-probe spectrometer (Light Conversion) pumped with a 10 kHz pulsed laser Pharos-SP (Light Conversion). The probe source was white light continuum generated by focusing 190 fs 1030 nm laser pulses in sapphire. Pump wavelength was set to 620 nm using an optical parametric amplifier Orpheus (Light Conversion).

The concentration of 10^{-3} M was selected for transient absorption measurements performed in toluene, $CHCl_3$, MeCN and DMSO.

Global analysis of transient absorption data

To fit transient absorption data global analysis was performed employing a sequential two excited state model displayed in **Figure S3** (a). The excited state decay was approximated with single-exponential function convoluted with 200 fs instrument response function (IRF). Reasonable fits with acceptable residual levels were obtained for all samples, example is displayed in **Figure S3** (b). Decay associated spectra (DAS) are displayed in **Figure S3** (c), while the normalized population dynamics of each excited state are shown in **Figure S3** (d).

II. UV-vis Spectroscopy in DMSO, CH_2Cl_2, and Toluene

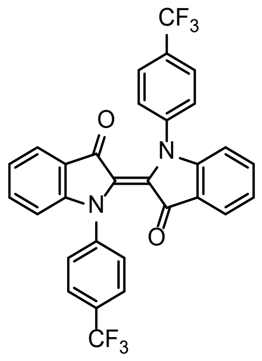

1

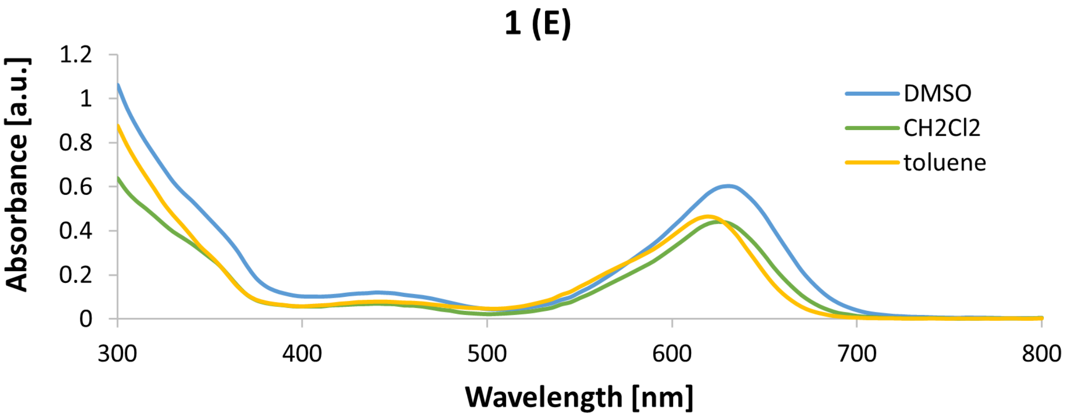

DMSO (c = 6.3 × 10^{-5} M, 25°C)

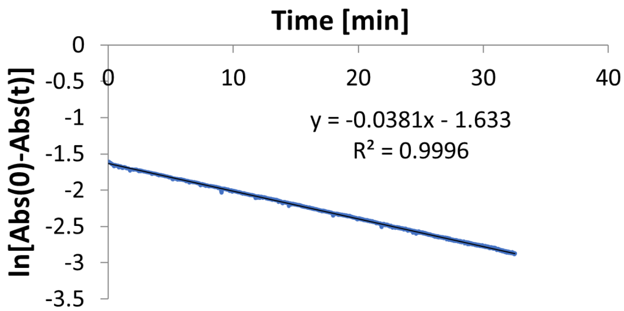

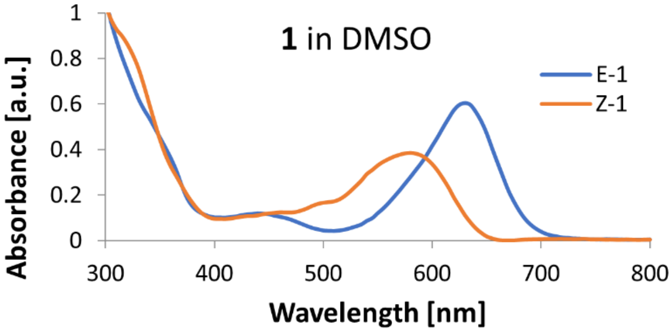

CH_2Cl_2 (c = 5.9 × 10^{-5} M, 25°C)

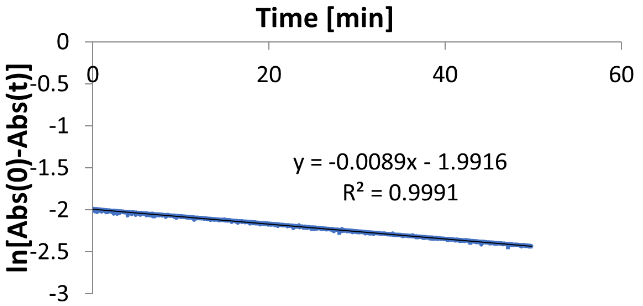

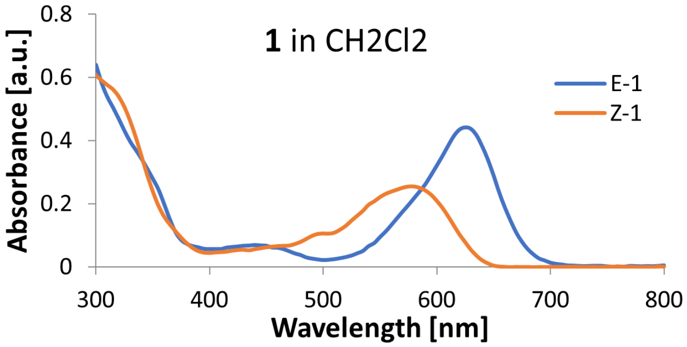

Toluene (c = 4.9 × 10^{-5} M, 25°C)

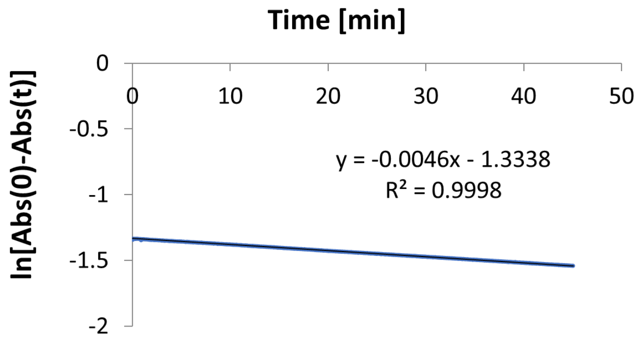

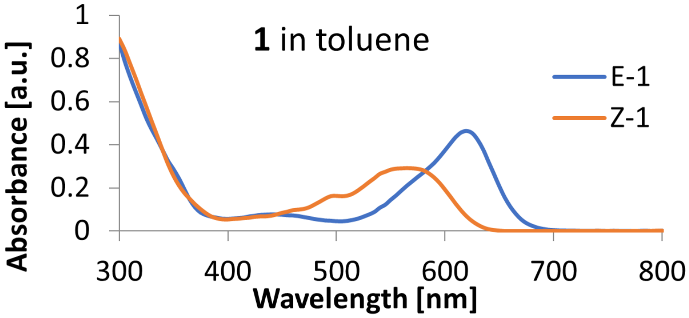

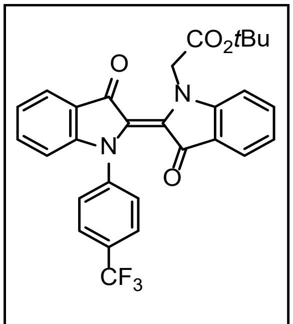

2

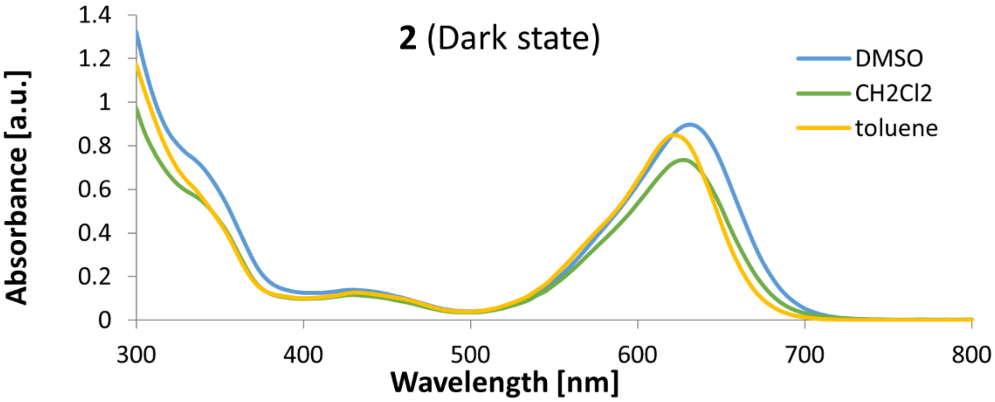

DMSO (c = 6.7 × 10^{-5} M, 25°C)

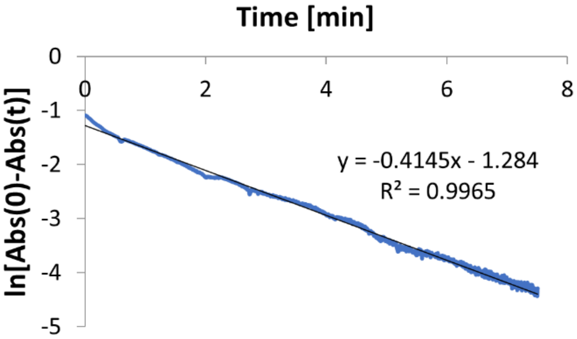

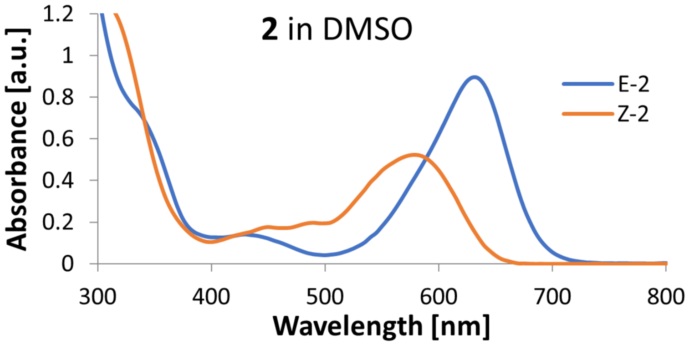

CH_2Cl_2 (c = 5.3 × 10^{-5} M, 25°C)

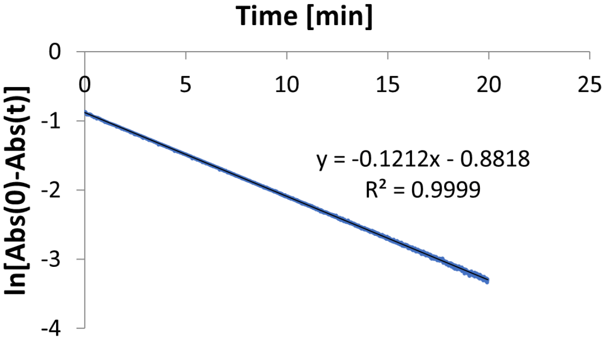

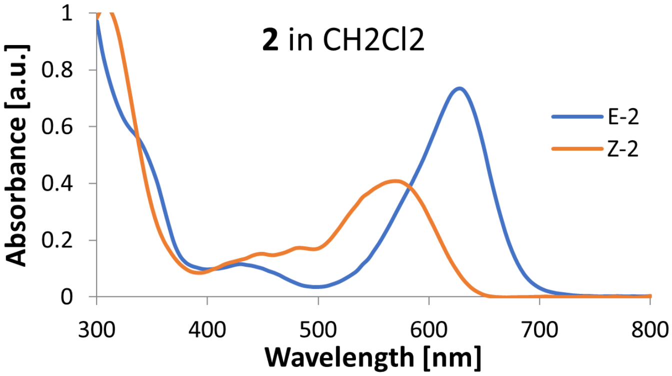

Toluene (c = 5.8 × 10^{-5} M, 25°C)

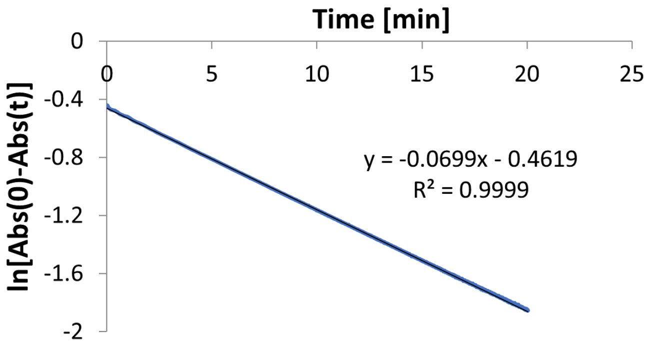

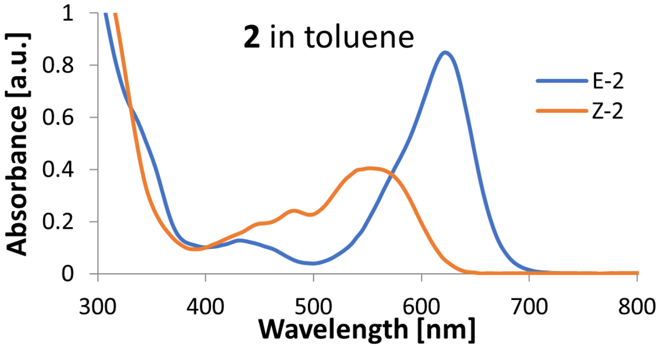

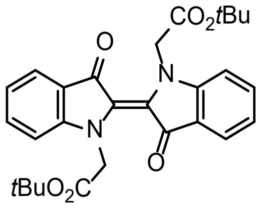

3

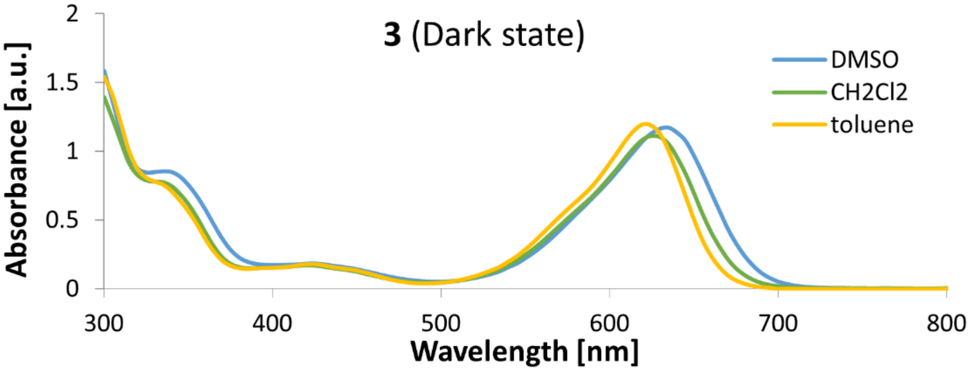

DMSO (c = 7.9 × 10^{-5} M, 25°C)

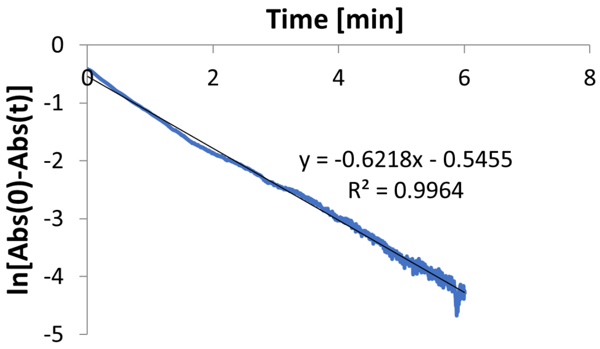

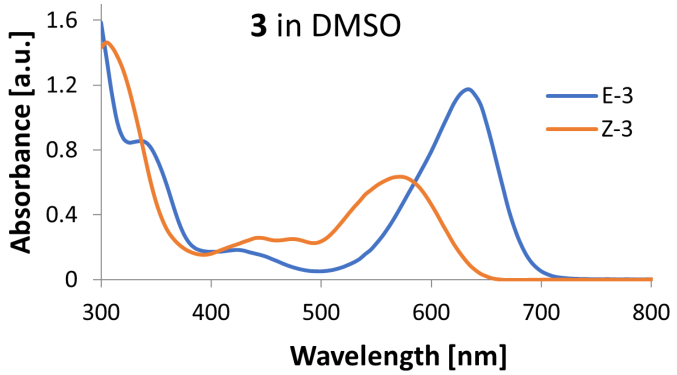

CH_2Cl_2 (c = 7.0 × 10^{-5} M, 25°C)

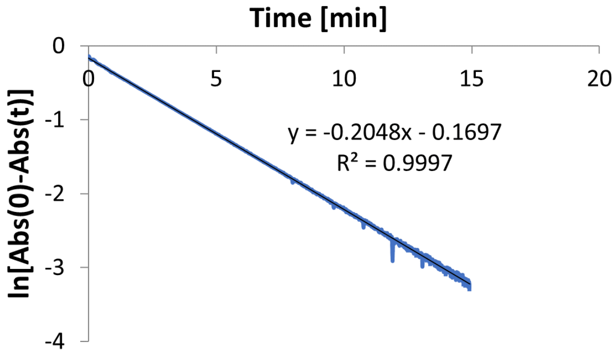

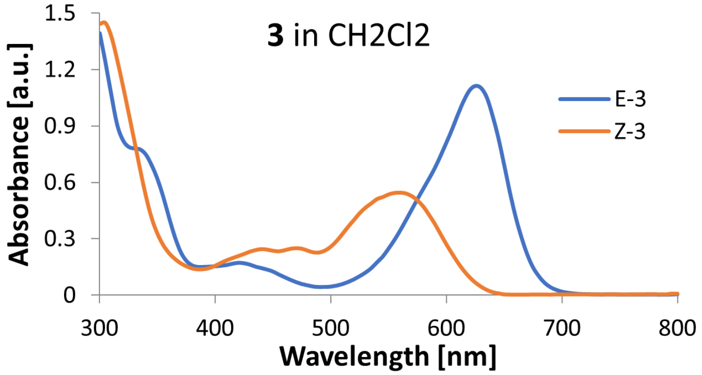

Toluene (c = 7.3 × 10^{-5} M, 25°C)

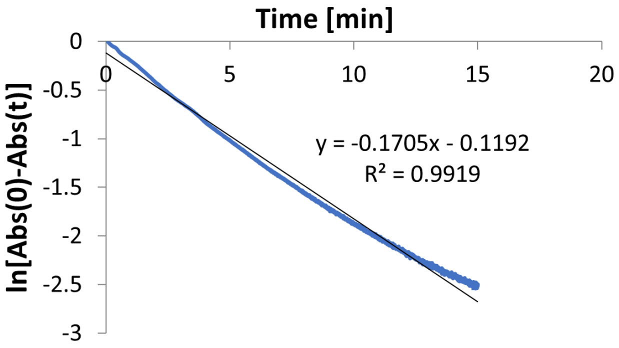

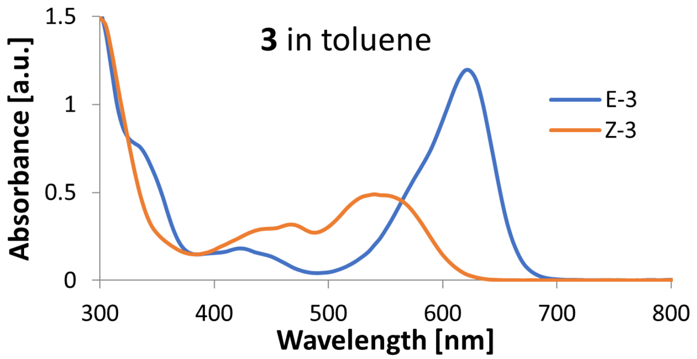

III. Linear Free Energy Analysis

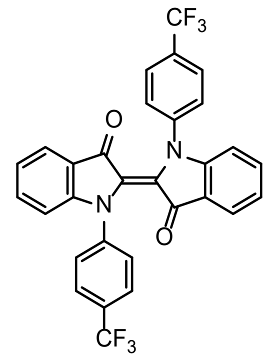

1

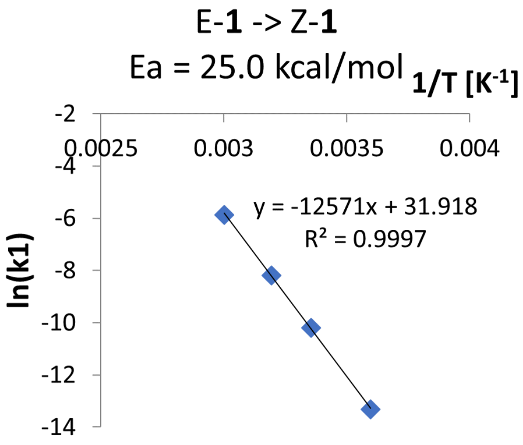

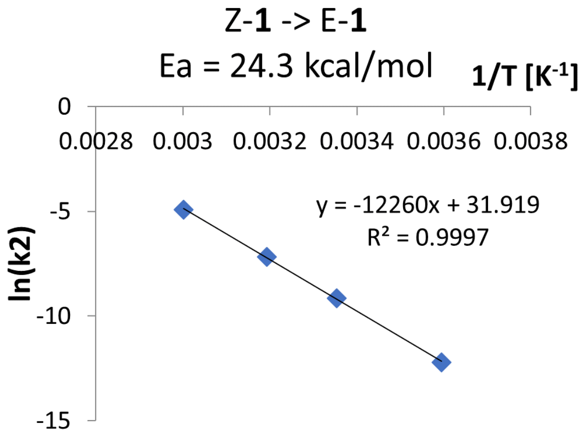

$[E]_{eq}/[Z]_{eq}$ were measured separately at different temperature: 60°C -> **2.55**; 40°C -> **2.71**; 25°C -> **2.84**; 5°C -> **3.07**

These values were then used for determining k_1 and k_2 from the formula: $K_{eq} = k_1/k_2 = [Z]_{eq}/[E]_{eq}$

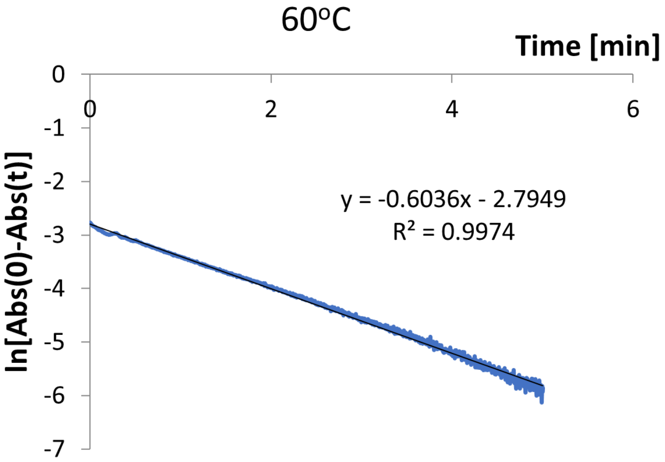

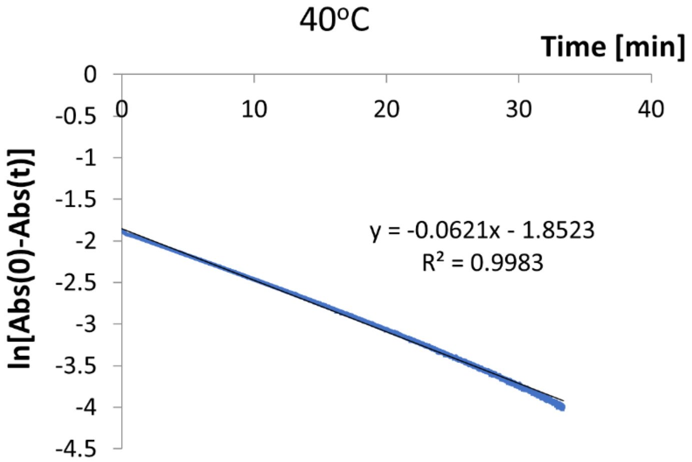

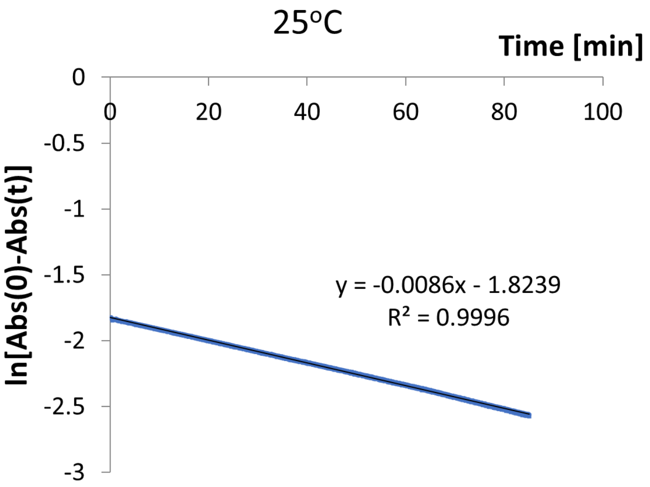

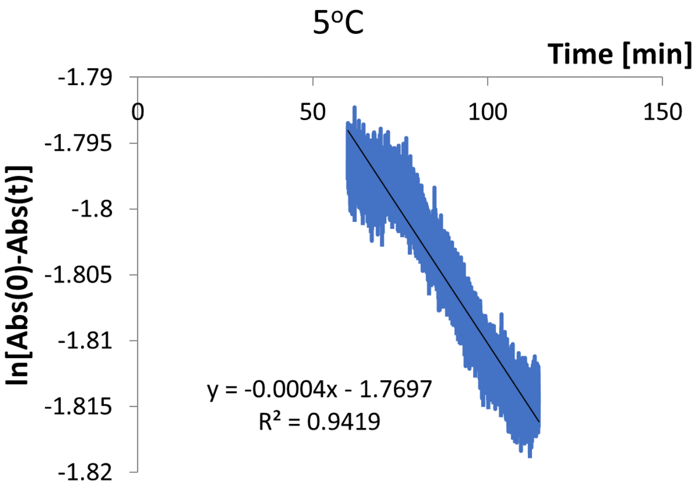

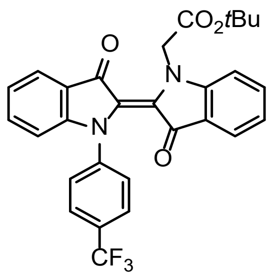
CO2tBu
O
N
N
O
CF3

2

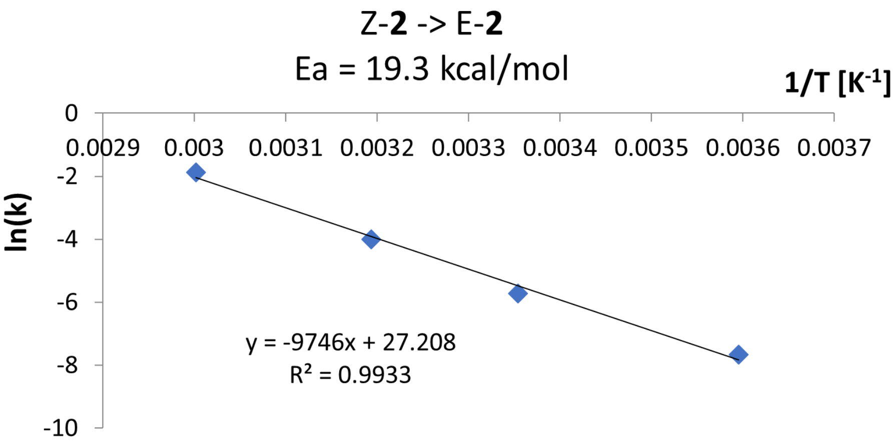
Z-2 -> E-2
Ea = 19.3 kcal/mol
1/T [K-1]
ln(k)
y = -9746x + 27.208
R² = 0.9933

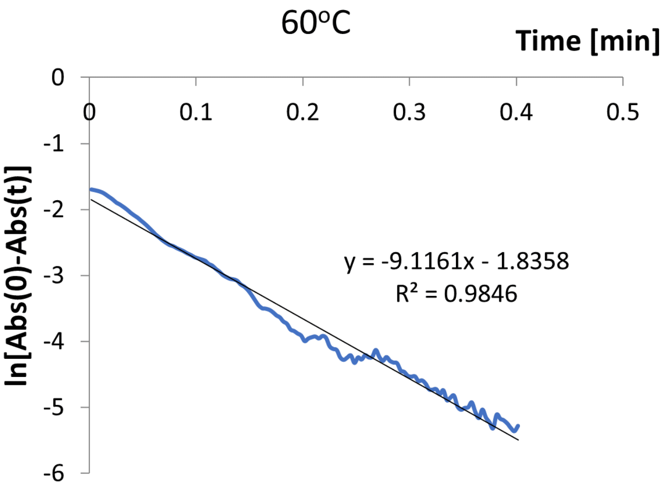
60oC
Time [min]
ln[Abs(0)-Abs(t)]
y = -9.1161x - 1.8358
R² = 0.9846

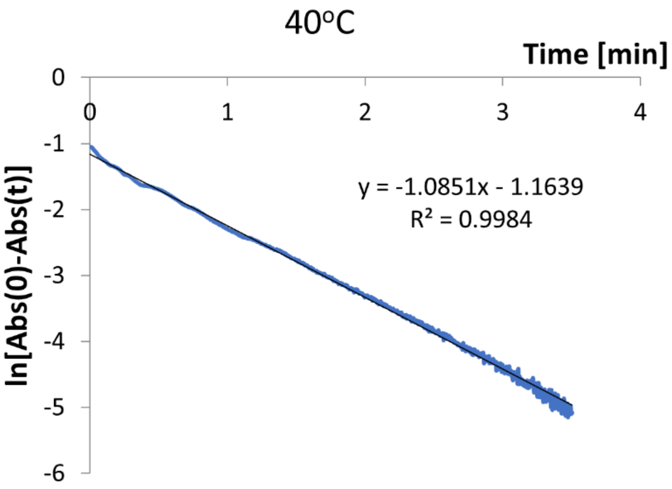
40oC
Time [min]
ln[Abs(0)-Abs(t)]
y = -1.0851x - 1.1639
R² = 0.9984

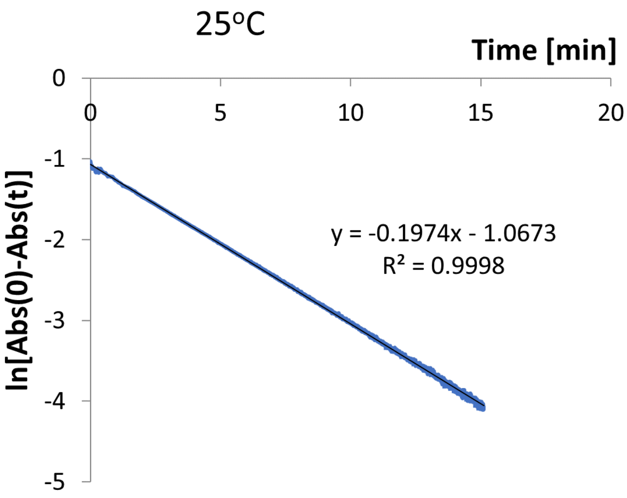
25oC
Time [min]
ln[Abs(0)-Abs(t)]
y = -0.1974x - 1.0673
R² = 0.9998

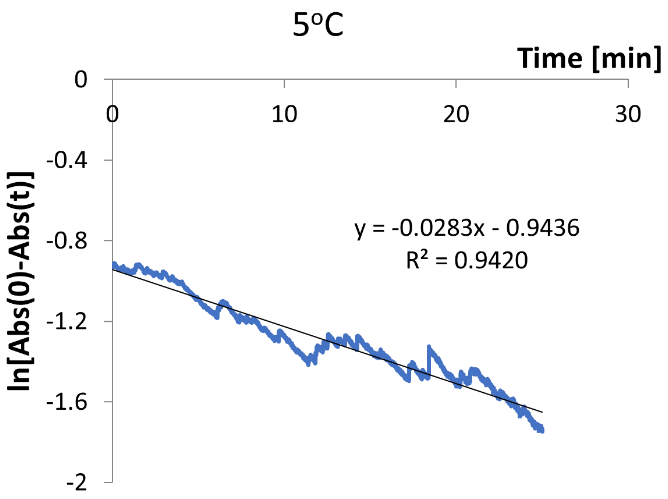
5oC
Time [min]
ln[Abs(0)-Abs(t)]
y = -0.0283x - 0.9436
R² = 0.9420

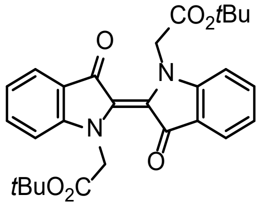

3

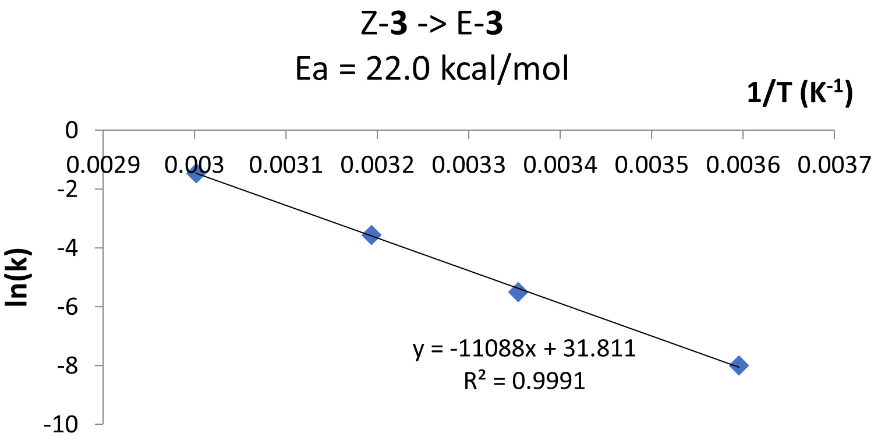
Z-3 -> E-3
Ea = 22.0 kcal/mol
1/T (K-1)
ln(k)
0
-2
-4
-6
-8
-10
0.0029 0.003 0.0031 0.0032 0.0033 0.0034 0.0035 0.0036 0.0037
y = -11088x + 31.811
R² = 0.9991

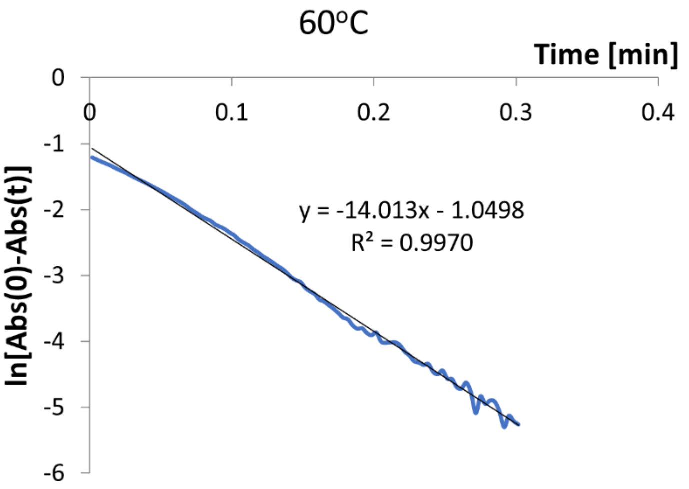
60°C
Time [min]
0 0.1 0.2 0.3 0.4
0 -1 -2 -3 -4 -5 -6
ln[Abs(0)-Abs(t)]
y = -14.013x - 1.0498
R² = 0.9970

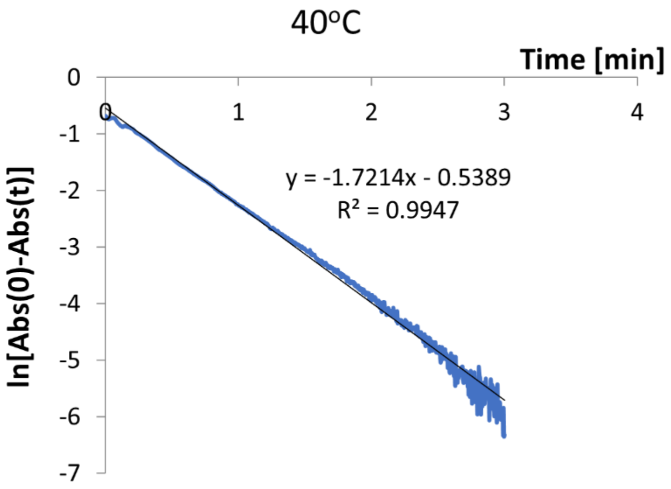
40°C
Time [min]
0 1 2 3 4
0 -1 -2 -3 -4 -5 -6 -7
ln[Abs(0)-Abs(t)]
y = -1.7214x - 0.5389
R² = 0.9947

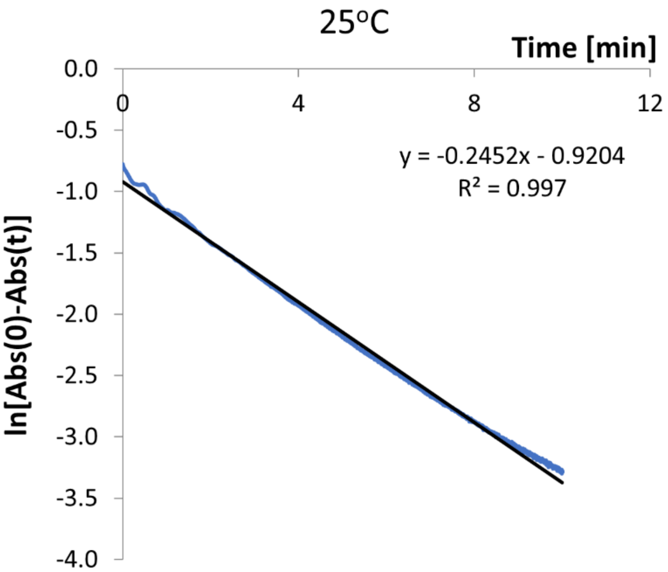
25°C
Time [min]
0 4 8 12
0.0 -0.5 -1.0 -1.5 -2.0 -2.5 -3.0 -3.5 -4.0
ln[Abs(0)-Abs(t)]
y = -0.2452x - 0.9204
R² = 0.997

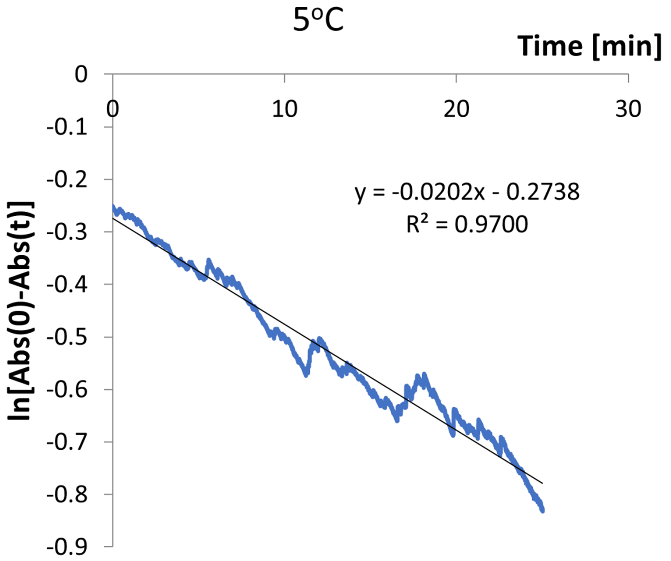
5°C
Time [min]
0 10 20 30
0 -0.1 -0.2 -0.3 -0.4 -0.5 -0.6 -0.7 -0.8 -0.9
ln[Abs(0)-Abs(t)]
y = -0.0202x - 0.2738
R² = 0.9700

Table S1. Activation energies in MeCN for thermal transformation from linear free energy analysis

Temperature		**1** (E→Z)	**1** (Z→E)	**2** (Z→E)	**3** (Z→E)
5°C	$t_{1/2}$ (min)	1733		24.5	34.3
	k (s^{-1})	1.6×10^{-6}	5.0×10^{-6}	4.7×10^{-4}	3.4×10^{-4}
25°C	$t_{1/2}$ (min)	80.6		3.5	2.8
	k (s^{-1})	3.7×10^{-5}	1.1×10^{-4}	3.3×10^{-3}	4.1×10^{-3}
40°C	$t_{1/2}$ (min)	11.2		0.64	0.40
	k (s^{-1})	2.8×10^{-4}	7.6×10^{-4}	1.8×10^{-2}	2.9×10^{-2}
60°C	$t_{1/2}$ (min)	1.1		0.08	0.05
	k (s^{-1})	2.8×10^{-3}	7.2×10^{-3}	1.5×10^{-1}	2.3×10^{-1}
Activation energy (kcal/mol)		25.0	24.3	19.3	22.0

IV. Photophysical Data

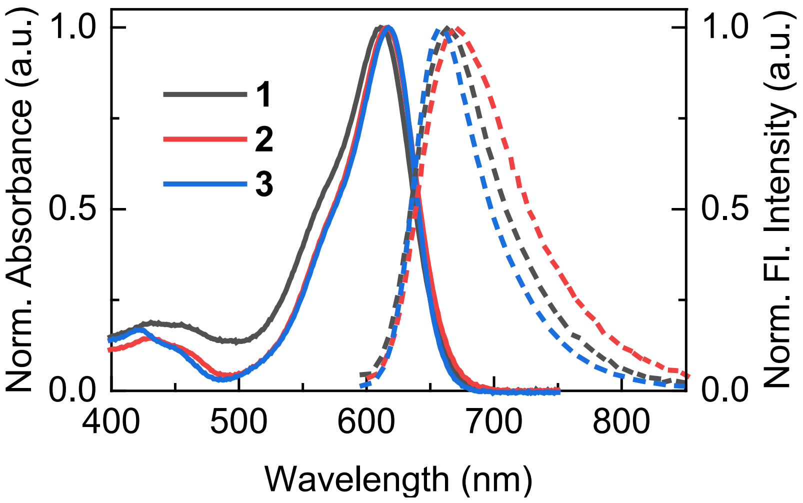

Figure S1. Absorption (solid line) and fluorescence (dashed line) spectra of compounds **1** (black), **2** (red) and **3** (blue) in 1% wt. Zeonex polymer films.

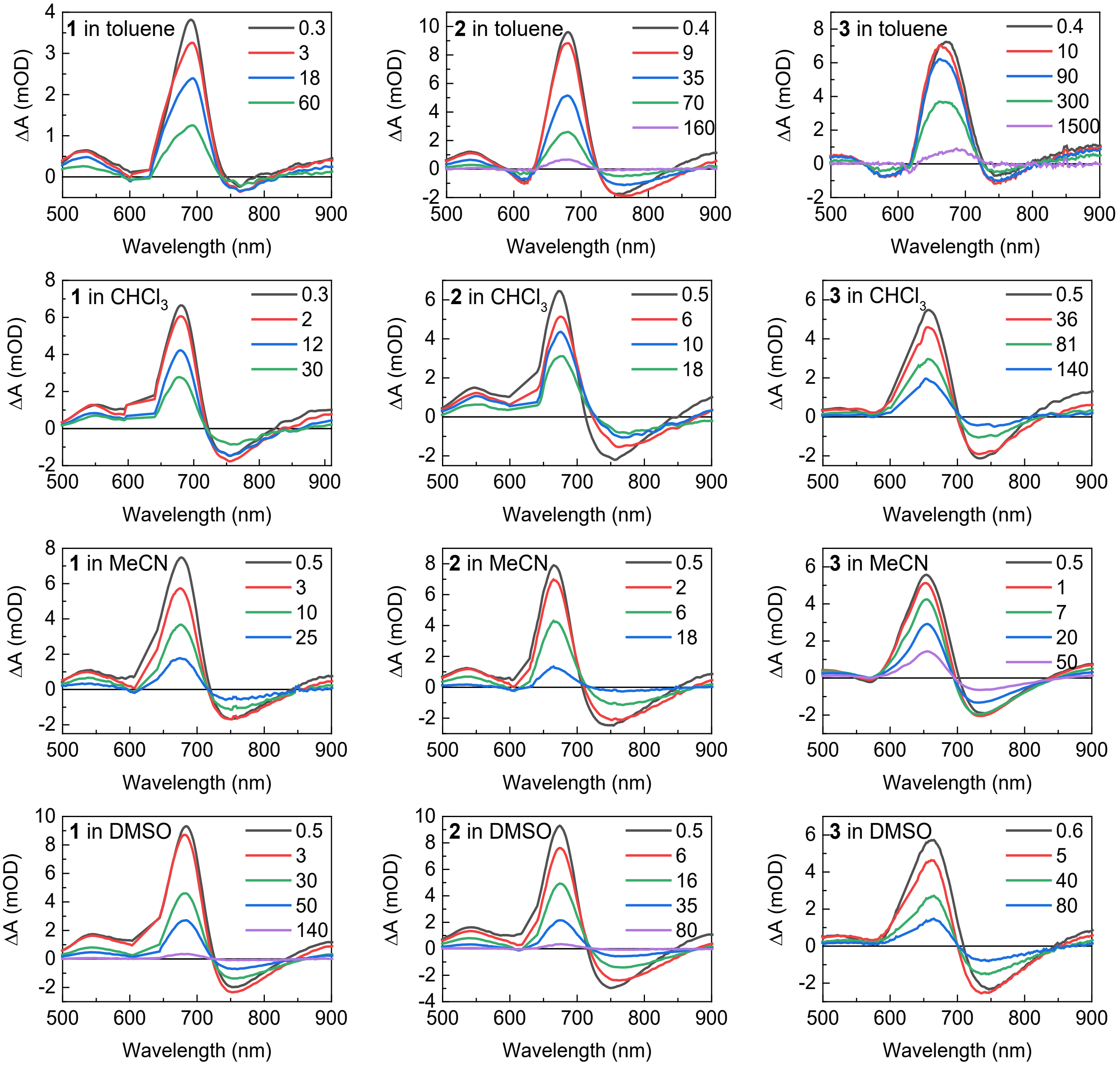

Figure S2. Transient absorption (TA) spectra of compounds **1**, **2** and **3** in toluene, $CHCl_3$, MeCN and DMSO at picosecond (ps) delay, indicated in legends.

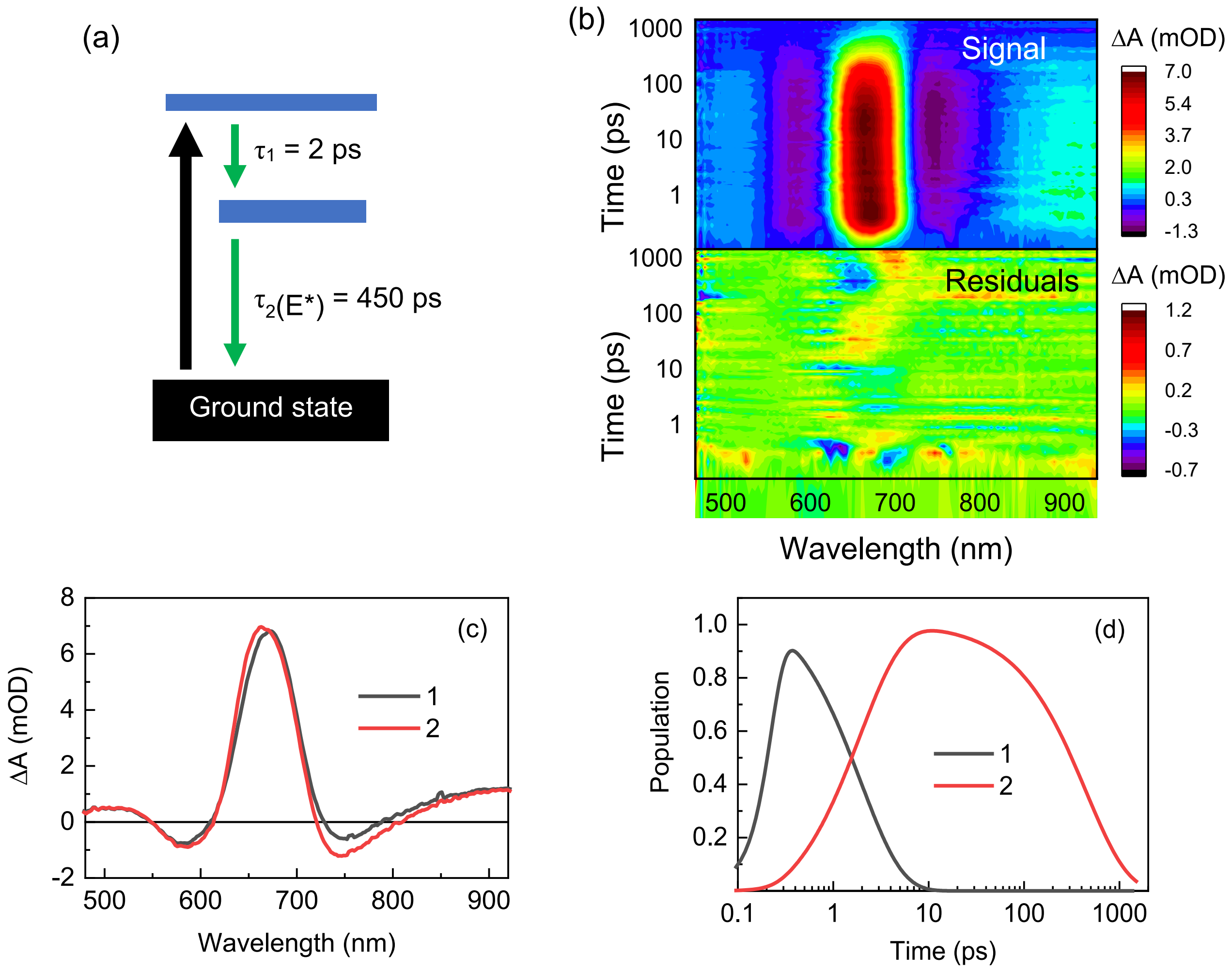

Figure S3. (a) Sequential global analysis model, corresponding fitted excited state lifetimes are indicated. (b) Transient absorption map and residuals of the global analysis of **3** in toluene. (c) Decay associated spectra (DAS) of each compartment. (d) Population dynamics of each compartment.

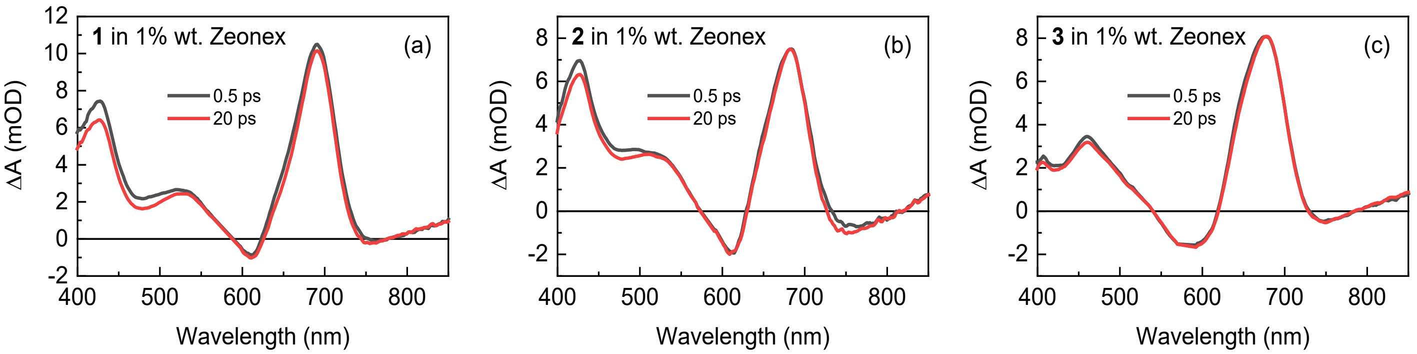

Figure S4. Transient absorption spectra of compounds **1**, **2** and **3** in 1% wt. Zeonex at 0.5 ps and 20 ps time delays.

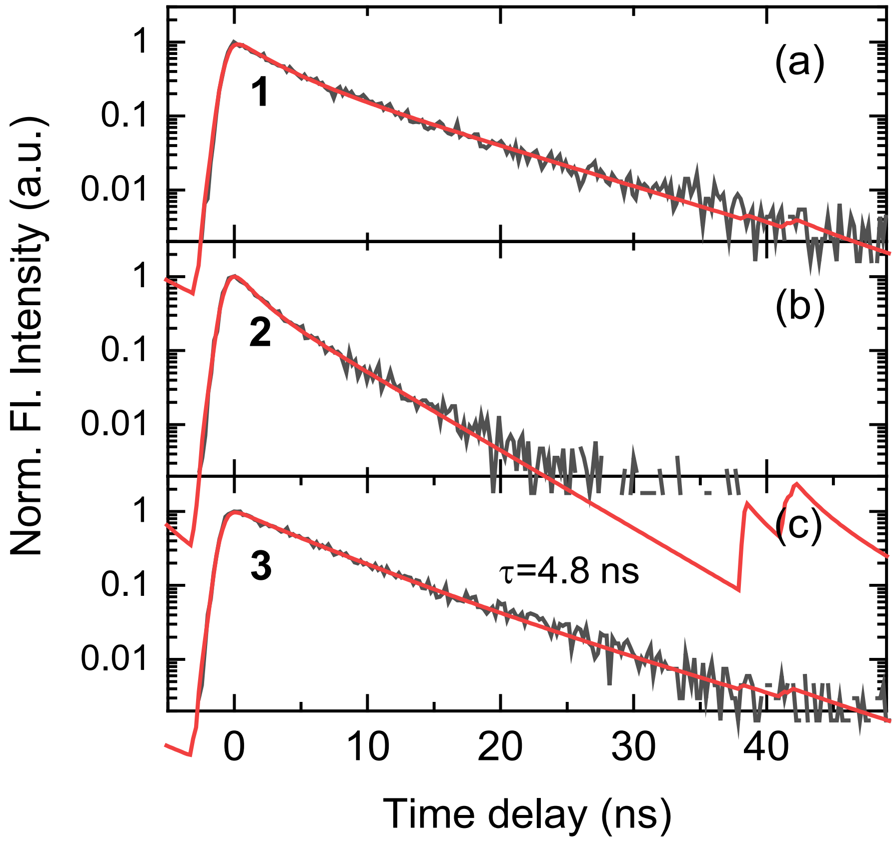

Figure S5. Fluorescence decay profiles of compounds **1** (a), **2** (b) and **3** (c) in 1% wt. Zeonex polymer films. Red lines correspond to exponential fits. Samples **1** and **2** showed non-exponential fluorescence decay, while fluorescence decay profile was mono-exponential for compound **3** with a time constant of 4.8 ns.

V. Computational Methods and Additional Data

Methods

For all our calculations the Gaussian16 program was used,[2] except for the spin-flip (SF) calculations that were performed with Gamess.[3,4] Our computational strategy comprised the following steps: i) the S_0 structures in their *E* and *Z* forms were optimized at the DFT level; ii) the S_1 excited state structures were optimized at the TD-DFT level, and, in cases the S_0-S_1 gap became smaller than 1.2 eV (> 1000 nm) we switched to SF-TD-PBE0,[5] see below for more details; iii) the vibrational frequencies on the structures optimized during the first two steps have been determined; iv) vertical TD-DFT transition energies to the singlet states were computed on both the ground-state and excited-state structures. We applied very tight energy (10^{-10} a.u.) convergence threshold and used the *ultrafine* integration DFT grid at all steps (including CPKS) during the Gaussian16 calculations. For the excited-state of the molecule **3** a further tightening of the integration grid to *superfine* was needed to obtain a structure without any residual imaginary frequencies. The non-spin-flip calculations rely on the range-separated hybrid ωB97X-D functional,[6] since we wished to simultaneously account for the dispersion interaction and reliably describe the possible charge transfer character of the excited-states. The PBE0 functional[7] was later used for single point calculations of absorption, emission energies and SF-TD-DFT as well, as this functional provide transition energies often reasonably fitting experiment for indigoids.[8] For the ground state activation barriers we found that the M06-2X[9] functional reproduces the experimental trends more faithfully than ωB97X-D and we therefore selected the former. Comparisons between the two functionals for this specific property can be found in Table S2. The optimization and vibrational calculations used the 6-31G(d) atomic basis set, whereas for the vertical TD-DFT calculations larger 6-311G(2d,p) basis set. During all steps, solvent effects (toluene, dichloromethane, acetonitrile, and DMSO) were accounted by the Polarizable Continuum Model (PCM).[10] For the TD-DFT part, the transition energies have been obtained using the cLR^2 approach,[11,12] that accounts for both linear-response and state-specific solvation effects in a consistent way.

In addition to these calculations we located transition state structures on the ground-state potential energy surface. Since the $Z \rightarrow E$ transformation of the investigated systems inevitably involves breaking the double bond we used broken symmetry DFT (BS-DFT) approach which allows α and β spin electrons to have different spatial distributions. At the located transition state structure, we always checked that the highest occupied spin-orbitals correspond to breaking central double bond. Conical intersections between the ground and the first excited-state were located for each dye using spin-flip TD-DFT (SF-TD-DFT) combined with, as stated above, the PBE0 functional and the 6-31G(d) basis set. The reference triplet state was obtained using restricted open shell (ROHF) formalism to avoid unwanted spin-contamination. The branching plane updating method[13] was used during the geometrical search.

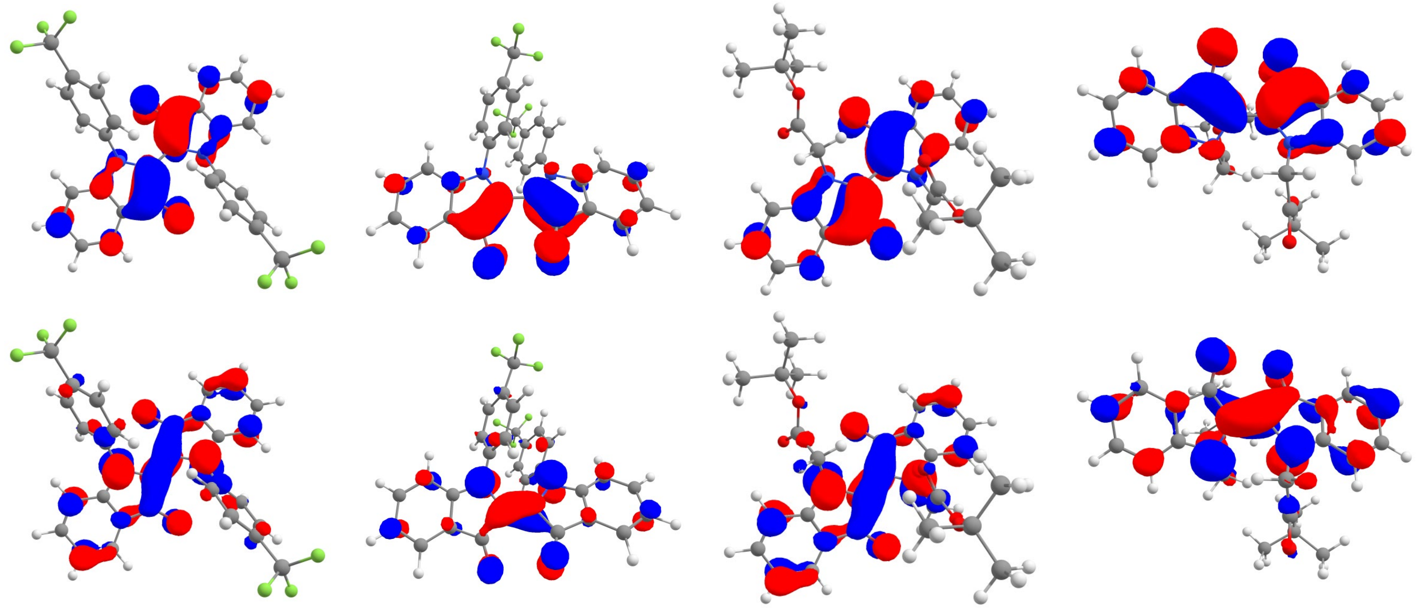

Figure S6. HOMO (top) and LUMO (bottom) orbitals of studied molecules **1** (first and second column) and **3** (third and fourth column) in both *E* and *Z* conformations.

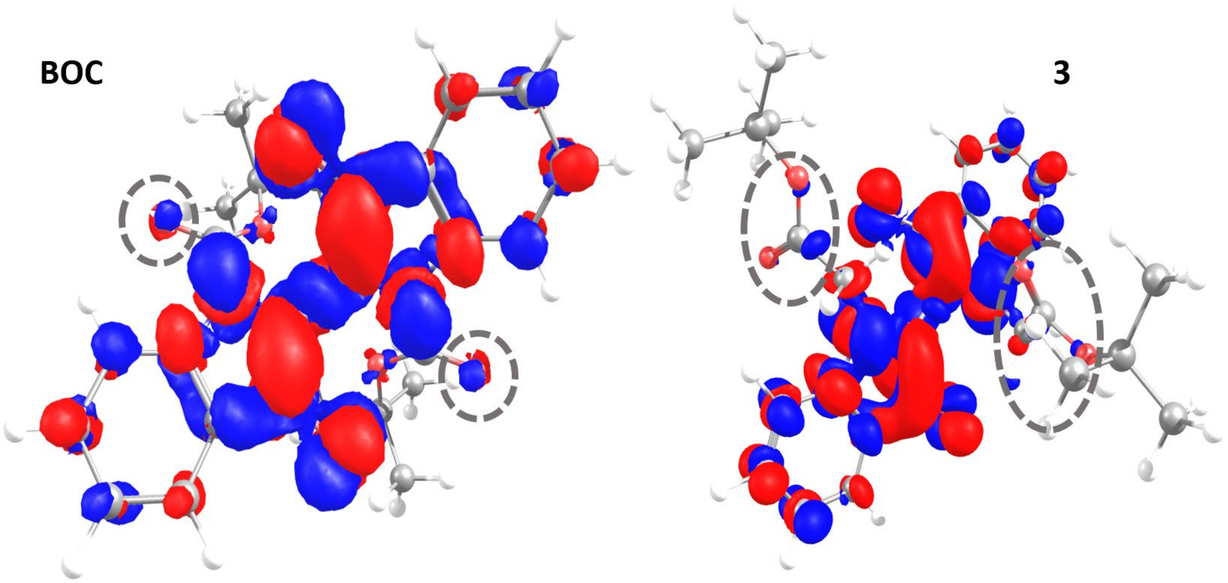

Figure S7. Electron density differences for the first excited state of **BOC-indigo** and compound **3**. A contour value of 0.0012 is applied.

Table S2. Comparison of activation energies calculated at ωB97X-D and M06-2X level of theory. Molecular geometry was always optimized at the same level of theory.

Compound	Solvent	E_a [kcal/mol] ωB97X-D/6-31G(d)	E_a [kcal/mol] M06-2X/6-31G(d)	E_a [kcal/mol] M06-2X/6-311+G(d,p)
1	Toluene	20.5	24.6	23.1
	DCM	20.5	24.5	22.9
	MeCN	20.5	24.4	22.8
	DMSO	20.5	24.3	22.8
2	Toluene	20.1	18.7	16.8
	DCM	20.2	19.3	16.6
	MeCN	20.3	19.4	16.5
	DMSO	20.3	19.4	16.4
3	Toluene	18.7	21.3	20.3
	DCM	18.3	20.5	20.8
	MeCN	18.1	20.1	20.8
	DMSO	18.0	20.1	20.8

Table S3. M06-2X/6-31G(d) dipole moments of the Z and TS structures in MeCN for the studied indigos in Debye.

Compound	Z	TS
1	3.9	5.8
2	9.2	8.1
3	5.8	11.0

Table S4. Vertical excitation wavelengths computed at the ωB97X-D/6-311G(2d,p) level. Experimental λ_{max} is given in parentheses. All values in nm.

Solvent/Compound	1		2		3	
	E	***Z***	***E***	***Z***	***E***	***Z***
Toluene	527(620)	495(565)	525(622)	478(551)	523(621)	491(540)
CH_2Cl_2	532(626)	503(577)	528(628)	486(569)	525(626)	495(560)
MeCN	532(628)	505(589)	528(622)	488(572)	525(623)	497(561)
DMSO	534(630)	507(580)	530(632)	490(580)	527(634)	498(573)

Table S5. Through-space charge transfer (CT) descriptors in Le Bahers' model for first excited states of studied molecules. Calculated at the ωB97XD/6-311G(2d,p) level of theory.

Compound/Descriptor	CT charge [e]	CT distance [Å]	H index [Å]	*t* index [Å]
**E-1**	0.26	0.61	1.76	-1.15
**Z-1**	0.22	1.75	1.45	0.30
**E-2**	0.26	1.01	1.94	-0.92
**Z-2**	0.21	1.29	2.00	-0.71
**E-3**	0.26	0.69	1.88	-1.19
**Z-3**	0.21	1.31	2.75	-1.43

Table S6. Dihedral angle CC=CC of the *E* structure optimized in the S_1 excited state and the MECI structure for studied molecules. Please note that due to computational limitations MECI structure is obtained in the gas phase.

Structure	Toluene	DCM	MeCN	DMSO
**E-ES-1**	128.1°	127.9°	127.8°	127.7°
**MECI-1**	100°			
**E-ES-2**	132.1°	130.7°	130.3°	130.2°
**MECI-2**	103 °			
**E-ES-3**	135.7°	130.7°	130.3°	129.9°
**MECI-3**	92.7°			

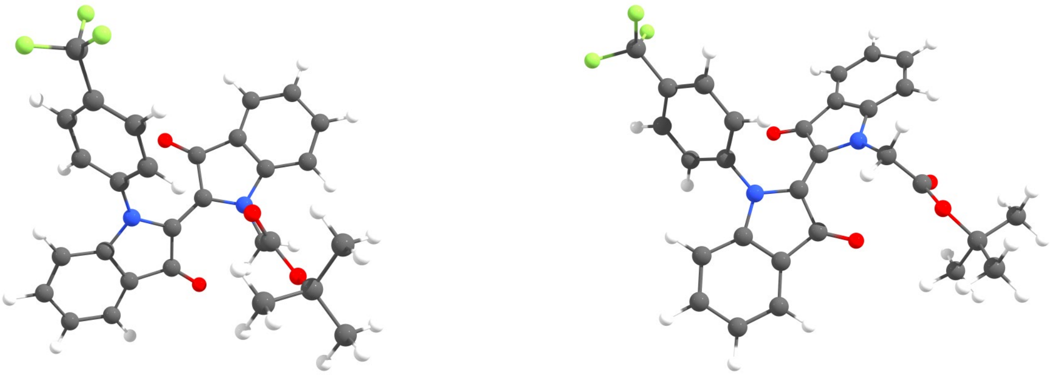

Figure S8. Schematic depiction of the global and nearby local minima excited state geometries in compound **2**.

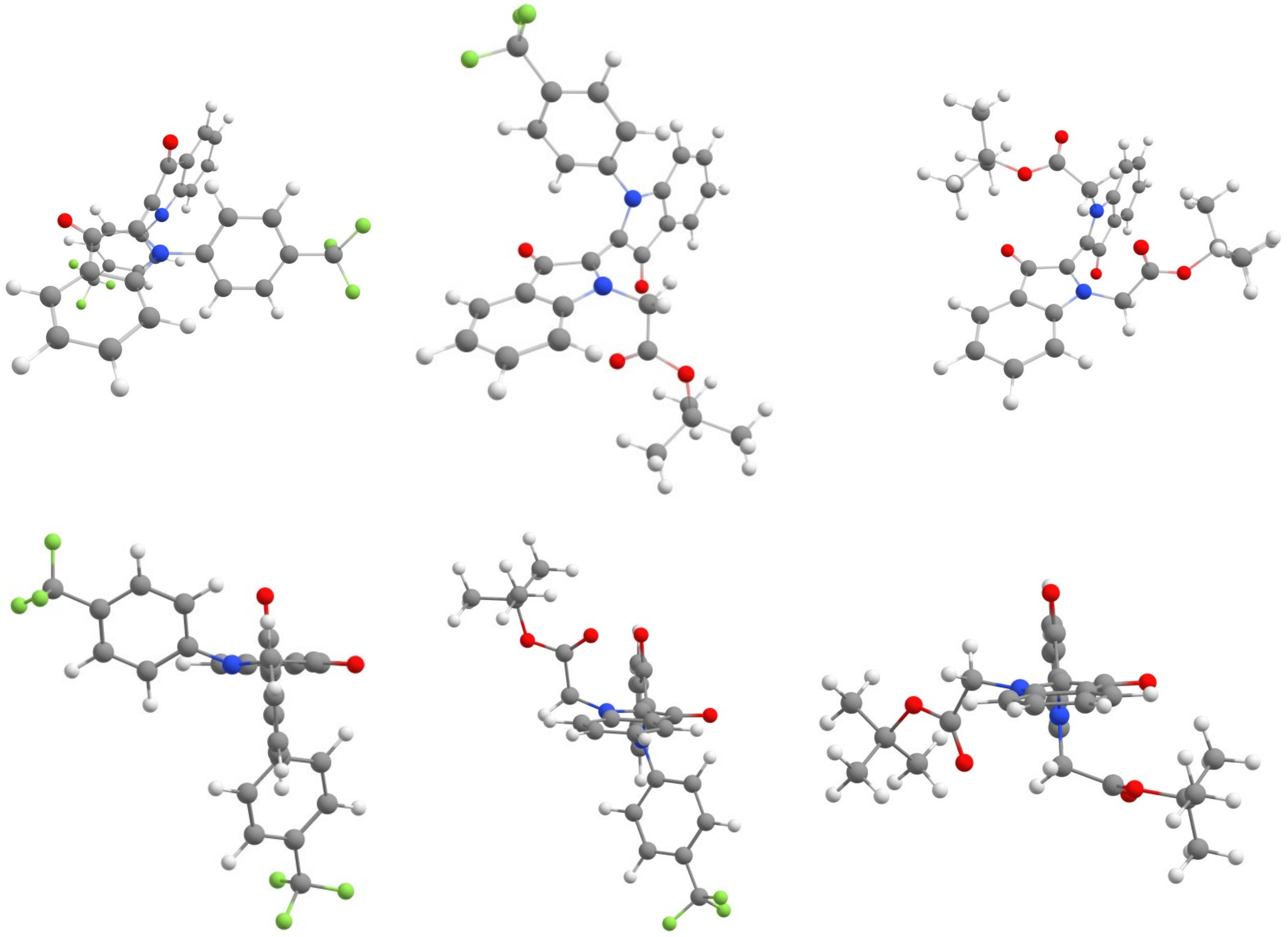

1 2 3

Figure S9. Minimum energy conical intersections between ground and first excited state. Upper and lower row offer different views.

VI. References

1. R. Sens and K. H. Drexhage, *J. Lumin.*, 1981, **24–25**, 709–712.
2. M. J. Frisch *et al.* Gaussian 16 Revision A.03, 2016, Gaussian Inc. Wallingford CT.
3. M. W. Schmidt, K. K. Baldridge, J. A. Boatz, S. T. Elbert, M. S. Gordon, J. H. Jensen, S. Koseki, N. Matsunaga, K. A. Nguyen, S. Su, T. L. Windus, M. Dupuis, J.A. Montgomery, *J. Comput. Chem.* 1993, **14**, 1347.
4. "Advances in electronic structure theory: GAMESS a decade later" M. S. Gordon, M. W. Schmidt pp. 1167-1189, in "Theory and Applications of Computational Chemistry: the first forty years" C. E. Dykstra, G. Frenking, K. S. Kim, G. E. Scuseria (editors), Elsevier, Amsterdam, 2005.
5. Y. Shao, M. Head-Gordon, A. I. Krylov, *J. Chem. Phys.* 2003, **118**, 4807.
6. J. D. Chai, M. Head-Gordon, *Phys. Chem. Chem. Phys.* 2008, **10**, 6615.
7. C. Adamo, V. Barone, *J. Chem. Phys.* 1999, **110**, 6158.
8. D. Jacquemin, J. Preat, V. Wathelet, M. Fontaine, E. A. Perpete, *JACS* 2006, **128**, 2072.
9. Y. Zhao, D. G. Truhlar, *Theor. Chem. Acc.* 2008, **120**, 215.
10. J. Tomasi, B. Mennucci, R. Cammi, *Chem. Rev.* 2005, **105**, 2999.
11. M. Caricato, B. Mennucci, J. Tomasi, F. Ingrosso, R. Cammi, S. Corni, G. Scalmani, *J. Chem. Phys.* 2006, **124**, 124520.
12. C. A. Guido, A. Chrayteh, G. Scalmani, B. Mennucci, D. Jacquemin, *J. Chem. Theory Comput.* 2021, **17**, 5155.
13. S. Maeda, K. Ohno, K. Morokuma, *J. Chem. Theor. Comput.* 2010, **6**, 1538.

VII. XYZ Coordinates

1-GS-Z M06-2X/6-31G(d) in acetonitrile

56

-2011.12841352

C	3.031907000	-2.941549000	-0.129691000
C	3.031855000	2.941591000	0.129684000
C	1.670380000	-2.870610000	0.162078000
C	1.670327000	2.870629000	-0.162072000
C	0.930246000	-4.029429000	0.382578000
C	0.930168000	4.029435000	-0.382558000
C	1.598955000	-5.247064000	0.258887000
C	1.598855000	5.247081000	-0.258866000
C	2.960010000	-5.324864000	-0.062678000
C	2.959912000	5.324906000	0.062685000
C	3.692312000	-4.158760000	-0.252296000
C	3.692238000	4.158814000	0.252288000
C	3.561952000	1.573041000	0.223808000
C	3.561978000	-1.572989000	-0.223827000
C	2.368071000	-0.680861000	0.059324000
C	2.368058000	0.680892000	-0.059331000
H	-0.119118000	-4.003899000	0.649420000
H	-0.119198000	4.003886000	-0.649389000
H	1.039475000	-6.163063000	0.420678000
H	1.039356000	6.163071000	-0.420645000
H	3.437858000	-6.293881000	-0.153871000
H	3.437743000	6.293931000	0.153879000
H	4.753484000	4.178496000	0.480619000
H	4.753556000	-4.178422000	-0.480638000
N	1.259509000	-1.520000000	0.228901000
N	1.259480000	1.520011000	-0.228899000

O	4.683437000	1.248240000	0.553011000
O	4.683451000	-1.248169000	-0.553047000
C	0.062237000	1.149340000	-0.897490000
C	0.105966000	0.297369000	-2.003229000
C	-1.158621000	1.635139000	-0.423263000
C	-1.079498000	-0.101294000	-2.607301000
C	-2.340136000	1.243562000	-1.036568000
C	-2.297150000	0.363258000	-2.115436000
H	1.061547000	-0.058328000	-2.374556000
H	-1.182359000	2.266284000	0.459176000
H	-1.056487000	-0.775704000	-3.456865000
H	-3.292509000	1.585393000	-0.645134000
C	-3.576659000	-0.179509000	-2.674053000
F	-4.587603000	0.694580000	-2.566480000
F	-3.464796000	-0.513345000	-3.967774000
F	-3.959250000	-1.294790000	-2.019725000
C	0.062263000	-1.149347000	0.897494000
C	0.105982000	-0.297372000	2.003231000
C	-1.158590000	-1.635161000	0.423269000
C	-1.079487000	0.101278000	2.607303000
C	-2.340109000	-1.243597000	1.036573000
C	-2.297134000	-0.363289000	2.115439000
H	1.061558000	0.058338000	2.374556000
H	-1.182321000	-2.266307000	-0.459170000
H	-1.056484000	0.775691000	3.456864000
H	-3.292479000	-1.585439000	0.645139000
C	-3.576649000	0.179464000	2.674054000
F	-4.587584000	-0.694636000	2.566483000
F	-3.464791000	0.513305000	3.967774000
F	-3.959253000	1.294740000	2.019723000

1-TS M06-2X/6-31G(d) in acetonitrile

56

-2011.08952838

C	-1.414035000	-2.646902000	-2.286665000
C	1.414035000	2.646915000	-2.286652000
C	-0.443120000	-2.863842000	-1.301807000
C	0.443119000	2.863850000	-1.301794000
C	-0.248225000	-4.107705000	-0.711917000
C	0.248222000	4.107710000	-0.711899000
C	-1.083442000	-5.139087000	-1.135591000
C	1.083438000	5.139095000	-1.135569000
C	-2.068456000	-4.936868000	-2.112995000
C	2.068453000	4.936881000	-2.112974000
C	-2.240022000	-3.687062000	-2.700407000
C	2.240022000	3.687077000	-2.700389000
C	1.324076000	1.234121000	-2.697998000
C	-1.324074000	-1.234107000	-2.698005000
C	-0.244184000	-0.686253000	-1.899141000
C	0.244185000	0.686263000	-1.899137000
H	0.517314000	-4.273507000	0.038223000
H	-0.517318000	4.273509000	0.038241000
H	-0.963445000	-6.124376000	-0.697299000
H	0.963439000	6.124382000	-0.697274000
H	-2.697969000	-5.767194000	-2.414402000
H	2.697965000	5.767209000	-2.414377000
H	2.992076000	3.517691000	-3.464567000
H	-2.992076000	-3.517672000	-3.464584000
N	0.254869000	-1.658066000	-1.084802000
N	-0.254869000	1.658072000	-1.084795000

O	2.025000000	0.614170000	-3.511088000
O	-2.024998000	-0.614152000	-3.511092000
C	-1.296197000	1.480898000	-0.131270000
C	-2.537686000	1.003759000	-0.552153000
C	-1.048910000	1.778066000	1.208202000
C	-3.541698000	0.813351000	0.388974000
C	-2.062277000	1.596240000	2.142074000
C	-3.300571000	1.111929000	1.728576000
H	-2.710088000	0.801592000	-1.604900000
H	-0.069960000	2.133570000	1.513254000
H	-4.515169000	0.449277000	0.078124000
H	-1.885708000	1.825115000	3.187095000
C	-4.381317000	0.857503000	2.740023000
F	-4.394461000	-0.425901000	3.137561000
F	-4.224697000	1.605630000	3.840835000
F	-5.598625000	1.119526000	2.241515000
C	1.296198000	-1.480897000	-0.131277000
C	2.537687000	-1.003760000	-0.552159000
C	1.048910000	-1.778069000	1.208194000
C	3.541700000	-0.813358000	0.388969000
C	2.062277000	-1.596249000	2.142066000
C	3.300573000	-1.111940000	1.728570000
H	2.710089000	-0.801588000	-1.604905000
H	0.069959000	-2.133572000	1.513245000
H	4.515172000	-0.449285000	0.078120000
H	1.885708000	-1.825127000	3.187086000
C	4.381318000	-0.857518000	2.740019000
F	5.598627000	-1.119532000	2.241508000
F	4.394456000	0.425882000	3.137569000
F	4.224701000	-1.605656000	3.840823000

1-GS-E M06-2X/6-31G(d) in acetonitrile

56

-2011.12270513

C	0.071165000	-2.979039000	-1.460413000
C	-0.071141000	2.979055000	-1.460381000
C	-1.255146000	-2.654449000	-1.161544000
C	1.255165000	2.654461000	-1.161493000
C	-2.227536000	-3.647825000	-1.074317000
C	2.227553000	3.647838000	-1.074237000
C	-1.826089000	-4.958344000	-1.327399000
C	1.826109000	4.958360000	-1.327309000
C	-0.502303000	-5.287820000	-1.649901000
C	0.502328000	5.287839000	-1.649830000
C	0.463025000	-4.290585000	-1.711041000
C	-0.462997000	4.290604000	-1.710999000
C	-0.866798000	1.748528000	-1.397611000
C	0.866822000	-1.748513000	-1.397613000
C	-0.125572000	-0.669356000	-1.047803000
C	0.125589000	0.669367000	-1.047794000
H	-3.255437000	-3.424249000	-0.814741000
H	3.255449000	3.424259000	-0.814646000
H	-2.568724000	-5.747947000	-1.269390000
H	2.568743000	5.747962000	-1.269278000
H	-0.236528000	-6.320921000	-1.843981000
H	0.236557000	6.320942000	-1.843902000
H	-1.501177000	4.512180000	-1.938696000
H	1.501208000	-4.512159000	-1.938722000
N	-1.386269000	-1.265607000	-0.952019000
N	1.386285000	1.265618000	-0.951982000

O	-2.044265000	1.592955000	-1.663548000
O	2.044294000	-1.592938000	-1.663528000
C	2.495493000	0.740674000	-0.230498000
C	2.310970000	0.157052000	1.022254000
C	3.772992000	0.846322000	-0.779819000
C	3.407341000	-0.329353000	1.722618000
C	4.870281000	0.376729000	-0.070138000
C	4.683895000	-0.214660000	1.177215000
H	1.315070000	0.083599000	1.446209000
H	3.895837000	1.268709000	-1.771694000
H	3.269288000	-0.790347000	2.694820000
H	5.865239000	0.449431000	-0.496128000
C	5.872792000	-0.682338000	1.962155000
F	6.377393000	0.295291000	2.736111000
F	5.564702000	-1.700227000	2.780112000
F	6.870196000	-1.093642000	1.164320000
C	-2.495489000	-0.740672000	-0.230548000
C	-2.310987000	-0.157065000	1.022214000
C	-3.772979000	-0.846312000	-0.779892000
C	-3.407370000	0.329333000	1.722566000
C	-4.870280000	-0.376727000	-0.070224000
C	-4.683914000	0.214647000	1.177140000
H	-1.315095000	-0.083617000	1.446187000
H	-3.895808000	-1.268686000	-1.771774000
H	-3.269333000	0.790315000	2.694775000
H	-5.865230000	-0.449423000	-0.496231000
C	-5.872824000	0.682317000	1.962066000
F	-6.377426000	-0.295316000	2.736017000
F	-5.564750000	1.700208000	2.780026000
F	-6.870222000	1.093613000	1.164220000

1-GS-E wB97XD/6-31G(d) ES optimized in acetonitrile

56

symmetry c1

C	-0.465877000	2.943929000	-1.519486000
C	0.465875000	-2.943931000	-1.519490000
C	0.834252000	2.822159000	-1.009668000
C	-0.834252000	-2.822161000	-1.009670000
C	1.624007000	3.935463000	-0.747421000
C	-1.624007000	-3.935465000	-0.747420000
C	1.084523000	5.184460000	-1.053362000
C	-1.084523000	-5.184462000	-1.053362000
C	-0.200475000	5.318022000	-1.592875000
C	0.200474000	-5.318024000	-1.592877000
C	-0.988980000	4.193956000	-1.826966000
C	0.988978000	-4.193958000	-1.826970000
C	1.046248000	-1.599422000	-1.634495000
C	-1.046251000	1.599420000	-1.634488000
C	0.003530000	0.704980000	-1.117574000
C	-0.003530000	-0.704982000	-1.117575000
H	2.615530000	3.852307000	-0.318116000
H	-2.615529000	-3.852309000	-0.318113000
H	1.679038000	6.072071000	-0.862901000
H	-1.679037000	-6.072073000	-0.862899000
H	-0.587204000	6.306769000	-1.816612000
H	0.587203000	-6.306771000	-1.816615000
H	1.994850000	-4.282218000	-2.225188000
H	-1.994853000	4.282216000	-2.225181000
N	1.106254000	1.459097000	-0.786713000
N	-1.106254000	-1.459099000	-0.786714000

O	2.136704000	-1.267055000	-2.103656000
O	-2.136707000	1.267053000	-2.103648000
C	-2.257108000	-0.983816000	-0.114460000
C	-2.123005000	-0.130520000	0.981430000
C	-3.522187000	-1.365261000	-0.565382000
C	-3.259043000	0.349089000	1.619422000
C	-4.651496000	-0.894271000	0.084269000
C	-4.520835000	-0.032176000	1.173508000
H	-1.141391000	0.148156000	1.344342000
H	-3.619259000	-1.992281000	-1.444388000
H	-3.155056000	1.015112000	2.468437000
H	-5.634321000	-1.179422000	-0.273828000
C	-5.748569000	0.438348000	1.901975000
F	-6.121421000	-0.433779000	2.859159000
F	-5.553047000	1.621107000	2.508397000
F	-6.799383000	0.578897000	1.075884000
C	2.257108000	0.983815000	-0.114460000
C	2.123006000	0.130515000	0.981428000
C	3.522187000	1.365265000	-0.565379000
C	3.259045000	-0.349092000	1.619420000
C	4.651497000	0.894276000	0.084272000
C	4.520837000	0.032178000	1.173508000
H	1.141393000	-0.148164000	1.344337000
H	3.619258000	1.992289000	-1.444381000
H	3.155059000	-1.015118000	2.468432000
H	5.634321000	1.179432000	-0.273822000
C	5.748571000	-0.438345000	1.901976000
F	6.121415000	0.433777000	2.859167000
F	5.553052000	-1.621110000	2.508389000
F	6.799388000	-0.578882000	1.075887000

1-MECI SF-PBE0/6-31G(d) in gas phase

56

symmetry c1

C	0.320963601	0.149023049	0.654845107
C	0.571152507	0.348059044	-5.329733680
C	0.949999595	1.397648594	0.533247240
C	1.747863959	-0.263531688	-4.897416117
C	1.280023340	2.157309789	1.655722214
C	2.633690899	-0.874539041	-5.764256710
C	1.018912625	1.601458494	2.903507992
C	2.305781686	-0.833923111	-7.124570280
C	0.429285592	0.335764535	3.037432520
C	1.140116752	-0.214929822	-7.577758298
C	0.069205274	-0.394392016	1.911450672
C	0.252130656	0.380257393	-6.678235771
C	-0.132066322	0.832348900	-4.130057534
C	0.043855922	-0.381800052	-0.690695262
C	0.512569830	0.649328577	-1.557204069
C	0.756903261	0.458785890	-2.968383290
H	1.721431256	3.145121977	1.569470365
H	3.532847360	-1.370298740	-5.414799449
H	1.283322640	2.164613479	3.794528376
H	2.974977670	-1.303483467	-7.839352221
H	0.249160399	-0.068164595	4.029716786
H	0.918271316	-0.205738826	-8.640463114
H	-0.668424194	0.849852581	-7.011591850
H	-0.406601813	-1.368250564	1.986447599
N	1.101569321	1.695983780	-0.823646882
N	1.813600728	-0.173466933	-3.473482963

O	-1.189274614	1.426286302	-4.041096586
O	-0.387445250	-1.518176557	-0.988129728
C	2.858123737	-0.801192406	-2.721946229
C	2.529811594	-1.843829799	-1.859867568
C	4.175382367	-0.383732929	-2.904356833
C	3.552200906	-2.469208017	-1.156086698
C	5.187266746	-1.022071132	-2.197633225
C	4.873329145	-2.062893194	-1.325098290
H	1.488300769	-2.137392117	-1.727759102
H	4.402834609	0.441624793	-3.572484467
H	3.315986600	-3.283665257	-0.479515608
H	6.219788555	-0.713749338	-2.325700070
C	5.966314057	-2.705646191	-0.516743072
F	6.222443562	-1.999511562	0.594495267
F	5.638738786	-3.948102676	-0.136982360
F	7.113255235	-2.781462401	-1.211531761
C	1.216542396	3.020360035	-1.295092140
C	2.350427150	3.775796535	-0.979607886
C	0.186347351	3.595196511	-2.050522819
C	2.441057588	5.100099025	-1.391656908
C	0.287726697	4.914477461	-2.465952246
C	1.409515978	5.674113650	-2.131354224
H	3.159470969	3.316330389	-0.419571203
H	-0.697210541	3.007108919	-2.277912305
H	3.316270580	5.689500944	-1.138345306
H	-0.525408530	5.368163417	-3.025103159
C	1.508334629	7.091155204	-2.609649249
F	2.406606391	7.798388243	-1.906047668
F	1.881742432	7.154989980	-3.901364159
F	0.328904663	7.728033647	-2.518313707

2-GS-Z M06-2X/6-31G(d) in acetonitrile

61

-1828.19841773

C	-1.426497000	3.757811000	-0.047925000
C	-2.010226000	2.615472000	0.500801000
C	-3.171887000	2.709418000	1.263158000
C	-3.691579000	3.984652000	1.482321000
C	-3.092459000	5.137594000	0.958717000
C	-1.949571000	5.026756000	0.176082000
C	-0.278865000	3.348694000	-0.872421000
C	-0.244043000	1.844200000	-0.752287000
H	-3.669405000	1.837533000	1.669647000
H	-4.593173000	4.080336000	2.079187000
H	-3.528321000	6.109974000	1.159255000
H	-1.470384000	5.895089000	-0.265752000
C	0.722315000	1.020326000	-1.246364000
C	1.522946000	1.239421000	-2.516645000
C	2.199317000	-0.049749000	-2.741207000
C	1.858837000	-0.908303000	-1.697121000
C	3.076346000	-0.440701000	-3.746807000
C	2.410685000	-2.182456000	-1.606320000
C	3.618898000	-1.719789000	-3.683177000
H	3.327477000	0.247368000	-4.548296000
C	3.288200000	-2.569367000	-2.618834000
H	2.172737000	-2.858180000	-0.791681000
H	4.303257000	-2.062535000	-4.451295000
H	3.726583000	-3.561599000	-2.577887000
O	0.506256000	4.074100000	-1.448147000
O	1.542641000	2.214222000	-3.238886000

N	0.947902000	-0.292303000	-0.819142000
N	-1.278467000	1.464004000	0.122875000
C	1.034390000	-0.622149000	0.588767000
H	0.298116000	-0.051564000	1.157125000
H	0.812254000	-1.680214000	0.748027000
C	2.412399000	-0.278687000	1.156029000
O	3.209933000	0.426621000	0.584468000
O	2.559655000	-0.847620000	2.346243000
C	3.783222000	-0.660147000	3.134856000
C	4.975224000	-1.230091000	2.373386000
C	3.958299000	0.815425000	3.476440000
C	3.504251000	-1.475881000	4.390568000
H	4.774714000	-2.266043000	2.083214000
H	5.194315000	-0.643403000	1.480425000
H	5.853231000	-1.219567000	3.025917000
H	3.052936000	1.200686000	3.955223000
H	4.789989000	0.921758000	4.179151000
H	4.173747000	1.407511000	2.586204000
H	4.360577000	-1.415190000	5.067496000
H	2.620678000	-1.091266000	4.907729000
H	3.333706000	-2.525341000	4.134359000
C	-1.909316000	0.186407000	0.093011000
C	-2.061123000	-0.508976000	-1.108139000
C	-2.362780000	-0.371919000	1.290850000
C	-2.676221000	-1.754256000	-1.109104000
C	-2.992330000	-1.610313000	1.284649000
C	-3.144894000	-2.299172000	0.084217000
H	-1.698201000	-0.075346000	-2.033965000
H	-2.195801000	0.153380000	2.225970000
H	-2.791054000	-2.300221000	-2.039490000

H	-3.340731000	-2.046893000	2.214236000
C	-3.857985000	-3.618806000	0.065009000
F	-5.173537000	-3.471760000	-0.169727000
F	-3.384132000	-4.428311000	-0.894341000
F	-3.743941000	-4.267623000	1.233466000

2-TS M06-2X/6-31G(d) in acetonitrile

61

-1828.16748622

C	-1.368129000	-2.757766000	1.127024000
C	-0.046411000	-2.801145000	0.667313000
C	0.610142000	-4.000220000	0.412578000
C	-0.120475000	-5.169879000	0.611010000
C	-1.449370000	-5.143788000	1.056769000
C	-2.082784000	-3.934515000	1.326976000
C	-1.721720000	-1.342617000	1.341354000
C	-0.531111000	-0.605231000	0.963164000
H	1.642077000	-4.032236000	0.081235000
H	0.357370000	-6.124782000	0.418275000
H	-1.982258000	-6.077780000	1.199071000
H	-3.105209000	-3.898771000	1.690714000
C	-0.400921000	0.844457000	0.922604000
C	-0.038013000	1.728974000	2.013305000
C	-0.200408000	3.086284000	1.460494000
C	-0.628821000	2.950892000	0.133034000
C	-0.005585000	4.349988000	2.008399000
C	-0.891777000	4.047521000	-0.680481000
C	-0.252748000	5.460411000	1.206252000
H	0.328333000	4.456453000	3.035775000
C	-0.691991000	5.305796000	-0.116672000

H	-1.261440000	3.932467000	-1.693386000
H	-0.111843000	6.458548000	1.606991000
H	-0.888839000	6.187524000	-0.717717000
O	-2.789517000	-0.864066000	1.747333000
O	0.349972000	1.385288000	3.140474000
N	-0.723114000	1.583614000	-0.171062000
N	0.440249000	-1.479274000	0.572278000
C	-1.097281000	1.054209000	-1.470826000
H	-0.682232000	1.701215000	-2.246022000
H	-0.687088000	0.049140000	-1.573746000
C	-2.620402000	1.038248000	-1.596029000
O	-3.254884000	2.032764000	-1.863448000
O	-3.095851000	-0.168376000	-1.333081000
C	-4.542920000	-0.429479000	-1.306392000
C	-5.206095000	0.448112000	-0.250702000
C	-5.118636000	-0.214448000	-2.701412000
C	-4.613339000	-1.896595000	-0.903887000
H	-4.668580000	0.344926000	0.696227000
H	-5.220739000	1.496468000	-0.551386000
H	-6.236740000	0.109333000	-0.108024000
H	-4.583104000	-0.829490000	-3.431035000
H	-6.169956000	-0.516989000	-2.702327000
H	-5.056607000	0.833444000	-2.999212000
H	-5.655876000	-2.226733000	-0.904846000
H	-4.049431000	-2.514680000	-1.608940000
H	-4.199246000	-2.026401000	0.099729000
C	1.758796000	-1.121348000	0.180786000
C	2.268461000	-1.598448000	-1.026690000
C	2.513129000	-0.286248000	1.006471000
C	3.555691000	-1.240884000	-1.409189000

C	3.793607000	0.078144000	0.609696000
C	4.310043000	-0.403073000	-0.591669000
H	1.654648000	-2.224817000	-1.665861000
H	2.099878000	0.059206000	1.949747000
H	3.962078000	-1.598611000	-2.348748000
H	4.390581000	0.730893000	1.237451000
C	5.717842000	-0.054306000	-0.981318000
F	5.888706000	-0.077865000	-2.310827000
F	6.065696000	1.166577000	-0.549424000
F	6.604918000	-0.917810000	-0.459135000

2-GS-E M06-2X/6-31G(d) in acetonitrile

61

-1828.20521031

C	0.439315000	3.745942000	-0.471710000
C	1.549320000	2.894230000	-0.475157000
C	2.817800000	3.383971000	-0.167393000
C	2.930092000	4.746020000	0.101455000
C	1.824704000	5.609284000	0.080517000
C	0.561440000	5.106130000	-0.199222000
C	-0.758209000	2.938444000	-0.716628000
C	-0.241256000	1.544891000	-0.916018000
H	3.688367000	2.740454000	-0.129129000
H	3.910516000	5.148291000	0.336766000
H	1.961150000	6.664109000	0.291052000
H	-0.319939000	5.740137000	-0.201305000
C	-0.950903000	0.483735000	-1.389595000
C	-0.391356000	-0.643929000	-2.225077000
C	-1.564241000	-1.447185000	-2.588793000
C	-2.685014000	-0.853316000	-2.003845000

C	-1.662701000	-2.617447000	-3.334550000
C	-3.944654000	-1.430140000	-2.135322000
C	-2.917431000	-3.196715000	-3.482870000
H	-0.774169000	-3.058295000	-3.776065000
C	-4.036511000	-2.603672000	-2.882888000
H	-4.831074000	-0.994453000	-1.688265000
H	-3.035473000	-4.107697000	-4.058954000
H	-5.009980000	-3.068582000	-3.003301000
O	-1.914948000	3.318409000	-0.823317000
O	0.763806000	-0.780013000	-2.580397000
N	-2.335221000	0.334453000	-1.327739000
N	1.155067000	1.577369000	-0.780300000
C	-3.074943000	0.699842000	-0.138643000
H	-2.915477000	1.751782000	0.099921000
H	-4.145185000	0.580431000	-0.320558000
C	-2.670341000	-0.174152000	1.046726000
O	-1.835177000	-1.048326000	0.979721000
O	-3.360689000	0.179607000	2.122060000
C	-3.164528000	-0.494145000	3.411825000
C	-3.558943000	-1.961658000	3.289146000
C	-1.723653000	-0.313853000	3.878718000
C	-4.125233000	0.250151000	4.330065000
H	-4.577570000	-2.046626000	2.898858000
H	-2.875904000	-2.502640000	2.632830000
H	-3.531166000	-2.422131000	4.281154000
H	-1.447904000	0.744731000	3.847961000
H	-1.640172000	-0.660129000	4.913124000
H	-1.028028000	-0.884946000	3.262187000
H	-4.078370000	-0.175630000	5.335858000
H	-3.858347000	1.309336000	4.384936000

H	-5.150773000	0.163741000	3.960243000
C	1.943037000	0.463801000	-0.374444000
C	1.458045000	-0.433645000	0.578060000
C	3.212368000	0.291336000	-0.926699000
C	2.250566000	-1.501289000	0.979804000
C	4.009465000	-0.766351000	-0.508541000
C	3.525945000	-1.661723000	0.442643000
H	0.460405000	-0.309275000	0.987655000
H	3.555812000	0.967372000	-1.702727000
H	1.876559000	-2.205424000	1.715588000
H	4.994865000	-0.907997000	-0.939791000
C	4.406861000	-2.775861000	0.921812000
F	5.209341000	-2.382841000	1.927643000
F	3.697196000	-3.818157000	1.380004000
F	5.210660000	-3.232657000	-0.051708000

2-ES-E wB97XD/6-31G(d) ES optimized in acetonitrile

61

symmetry c1

C	-0.672967000	3.463526000	-1.285190000
C	0.590373000	3.077290000	-0.816219000
C	1.518188000	4.010985000	-0.368195000
C	1.159797000	5.355849000	-0.452221000
C	-0.085647000	5.755643000	-0.951906000
C	-1.017222000	4.807466000	-1.366982000
C	-1.437429000	2.253623000	-1.607874000
C	-0.529063000	1.147715000	-1.288656000
H	2.480089000	3.721271000	0.037602000
H	1.866764000	6.107410000	-0.116161000
H	-0.329440000	6.811812000	-1.000007000

H	-1.996830000	5.099007000	-1.732396000
C	-0.683208000	-0.225070000	-1.580740000
C	0.321859000	-1.105988000	-2.216425000
C	-0.332233000	-2.417827000	-2.268438000
C	-1.627539000	-2.276832000	-1.747264000
C	0.102464000	-3.646769000	-2.749930000
C	-2.507572000	-3.349576000	-1.669068000
C	-0.768199000	-4.732331000	-2.677096000
H	1.102180000	-3.752197000	-3.159553000
C	-2.053035000	-4.580867000	-2.140379000
H	-3.508359000	-3.252167000	-1.262830000
H	-0.448704000	-5.706378000	-3.032819000
H	-2.714762000	-5.439424000	-2.088928000
O	-2.583689000	2.173032000	-2.068160000
O	1.430991000	-0.771050000	-2.639928000
N	-1.824846000	-0.945093000	-1.369512000
N	0.667193000	1.673074000	-0.836802000
C	-2.943263000	-0.501413000	-0.572861000
H	-3.155420000	0.544824000	-0.801412000
H	-3.833532000	-1.071983000	-0.846352000
C	-2.673586000	-0.676070000	0.919479000
O	-1.678564000	-1.205251000	1.365064000
O	-3.680119000	-0.162501000	1.603692000
C	-3.697717000	-0.144287000	3.077547000
C	-3.699625000	-1.574102000	3.609840000
C	-2.516441000	0.673636000	3.592396000
C	-5.017649000	0.553598000	3.385830000
H	-4.519333000	-2.145170000	3.162741000
H	-2.755602000	-2.079156000	3.399325000
H	-3.849343000	-1.550756000	4.693676000

H	-2.508593000	1.664292000	3.126909000
H	-2.617768000	0.803733000	4.674249000
H	-1.566589000	0.176270000	3.389422000
H	-5.148736000	0.630263000	4.469089000
H	-5.029113000	1.562027000	2.961617000
H	-5.858552000	-0.011435000	2.972688000
C	1.691661000	0.929522000	-0.207361000
C	1.355128000	-0.076990000	0.699320000
C	3.028426000	1.196984000	-0.513170000
C	2.366089000	-0.820965000	1.295967000
C	4.028885000	0.458775000	0.095817000
C	3.698078000	-0.554551000	0.998179000
H	0.317756000	-0.271939000	0.949150000
H	3.277637000	1.943522000	-1.258549000
H	2.107042000	-1.605663000	1.997678000
H	5.066372000	0.654261000	-0.151811000
C	4.795633000	-1.318465000	1.683660000
F	5.272749000	-0.649887000	2.752366000
F	4.380491000	-2.516421000	2.126938000
F	5.841464000	-1.533380000	0.865937000

2-MECI SF-PBE0/6-31G(d) in gas phase

61

symmetry c1

C	-0.998179557	3.751308821	-0.462161271
C	-1.637892837	2.745222832	0.277407184
C	-2.641989727	3.045053896	1.199017643
C	-2.946955990	4.387191928	1.398435683
C	-2.286745448	5.404522438	0.693365843
C	-1.312462964	5.089853859	-0.246555930

C	-0.060110731	3.128099299	-1.420385650
C	-0.258290532	1.723538344	-1.208220521
H	-3.177508641	2.267636596	1.734949747
H	-3.720916621	4.649126131	2.115234336
H	-2.552140428	6.441733146	0.878309988
H	-0.805175289	5.856397443	-0.825767132
C	0.628269776	0.670756986	-1.649576333
C	0.471381000	-0.268096155	-2.817941900
C	1.635957437	-1.164599745	-2.748128222
C	2.410171607	-0.750940239	-1.665304647
C	2.023626264	-2.248111282	-3.519049256
C	3.580514074	-1.391498278	-1.303506444
C	3.198832564	-2.915876322	-3.169699249
H	1.420459903	-2.558727358	-4.366423886
C	3.960615856	-2.491923350	-2.079598016
H	4.186283092	-1.070672374	-0.461762143
H	3.525305264	-3.773867580	-3.749092532
H	4.871521155	-3.025358045	-1.824428299
O	0.765508720	3.702053805	-2.151423666
O	-0.457690274	-0.274731434	-3.604437098
N	1.777639203	0.364482308	-1.053277997
N	-1.149352945	1.504681896	-0.137396537
C	2.474725543	1.221456423	-0.123004160
H	1.767775879	1.962352504	0.257509374
H	2.869102162	0.644557755	0.717986507
C	3.605371052	1.933717478	-0.879357614
O	3.716240066	1.888438380	-2.077320285
O	4.389714609	2.558078022	-0.011971278
C	5.525448767	3.368908217	-0.490846627
C	6.498283680	2.491622094	-1.271889696

C	5.001161132	4.536727443	-1.315112951
C	6.162142980	3.851195290	0.804225352
H	6.800046756	1.626442220	-0.670984731
H	6.060386660	2.141540876	-2.208041965
H	7.398276151	3.072152974	-1.501476672
H	4.283175244	5.123044489	-0.733108570
H	5.838830608	5.189057184	-1.584525524
H	4.513798244	4.197195146	-2.231277153
H	7.030639725	4.477050572	0.576778492
H	5.451305651	4.441551069	1.390421932
H	6.495459707	3.002929458	1.410987030
C	-1.846755896	0.297068847	0.067156041
C	-2.333579359	-0.430570260	-1.025941937
C	-2.060297086	-0.178289281	1.365821421
C	-3.015584824	-1.619962122	-0.817533272
C	-2.758591641	-1.362242372	1.567354637
C	-3.231756216	-2.090984287	0.477495199
H	-2.183158418	-0.046438272	-2.029765405
H	-1.658275571	0.373791482	2.209967503
H	-3.390664348	-2.180848541	-1.667949570
H	-2.915238053	-1.734454002	2.574832155
C	-4.016350584	-3.349979703	0.692452221
F	-5.336279771	-3.107676410	0.776768927
F	-3.843720393	-4.220916926	-0.315518845
F	-3.660724321	-3.970270057	1.830369574

3-GS-Z M06-2X/6-31G(d) in acetonitrile

66

-1645.27856906

C	-1.941515000	2.810691000	0.016492000

C	-2.474430000	1.637030000	-0.519693000
C	-3.851712000	1.448108000	-0.601352000
C	-4.664862000	2.466049000	-0.104559000
C	-4.136435000	3.637863000	0.453810000
C	-2.759008000	3.819338000	0.514384000
C	-0.475897000	2.737060000	-0.091766000
C	-0.213587000	1.404190000	-0.767485000
H	-4.286408000	0.548067000	-1.021777000
H	-5.742135000	2.340788000	-0.150670000
H	-4.806085000	4.401218000	0.834062000
H	-2.319297000	4.721970000	0.927573000
C	1.008034000	0.843709000	-0.970329000
C	2.311184000	1.571039000	-1.193381000
C	3.274888000	0.498804000	-1.496327000
C	2.606558000	-0.722222000	-1.432836000
C	4.637698000	0.569472000	-1.767139000
C	3.288378000	-1.922046000	-1.611647000
C	5.329531000	-0.620404000	-1.963208000
H	5.134752000	1.533927000	-1.810602000
C	4.654284000	-1.846191000	-1.880145000
H	2.788288000	-2.882850000	-1.555967000
H	6.392465000	-0.606478000	-2.177661000
H	5.208565000	-2.767215000	-2.031273000
O	0.338105000	3.519985000	0.353746000
O	2.517847000	2.768334000	-1.210007000
N	1.234120000	-0.537921000	-1.154722000
N	-1.452374000	0.777657000	-0.948277000
C	0.624844000	-1.463100000	-0.211255000
H	-0.447594000	-1.278343000	-0.136233000
H	0.753063000	-2.491264000	-0.557384000

C	-1.724666000	-0.197081000	-1.987527000
H	-0.785824000	-0.505658000	-2.452936000
H	-2.344597000	0.249667000	-2.774863000
C	1.139241000	-1.358674000	1.225344000
O	0.742587000	-2.104956000	2.092486000
O	1.992877000	-0.357304000	1.363998000
C	-2.435954000	-1.467417000	-1.521068000
O	-2.709703000	-2.350917000	-2.298920000
O	-2.682784000	-1.452952000	-0.216643000
C	2.511412000	0.072473000	2.663906000
C	1.350619000	0.481784000	3.563335000
C	3.362759000	-1.037335000	3.267513000
C	3.358866000	1.282088000	2.288774000
H	0.725378000	1.220936000	3.052157000
H	0.738108000	-0.377457000	3.840478000
H	1.748773000	0.938025000	4.474306000
H	4.132740000	-1.348458000	2.554901000
H	3.857076000	-0.660890000	4.167903000
H	2.752062000	-1.900846000	3.535961000
H	3.804204000	1.718613000	3.186920000
H	4.160390000	0.986946000	1.604326000
H	2.738893000	2.038191000	1.795590000
C	-3.268510000	-2.604341000	0.481331000
C	-4.659314000	-2.892769000	-0.070857000
C	-2.325492000	-3.796592000	0.366435000
C	-3.340146000	-2.112733000	1.921028000
H	-5.269557000	-1.984356000	-0.045773000
H	-4.612749000	-3.266659000	-1.094185000
H	-5.141609000	-3.645991000	0.559007000
H	-1.344060000	-3.540977000	0.780303000

H	-2.732940000	-4.628841000	0.948037000
H	-2.213972000	-4.118160000	-0.670586000
H	-3.763017000	-2.894228000	2.558032000
H	-2.337683000	-1.866206000	2.284464000
H	-3.971486000	-1.221903000	1.988926000

3-TS M06-2X/6-31G(d) in acetonitrile

66

-1645.24652547

C	-0.812830000	2.643811000	-1.470113000
C	-1.855330000	1.709142000	-1.427432000
C	-3.193647000	2.085709000	-1.440977000
C	-3.459425000	3.451596000	-1.509393000
C	-2.428458000	4.400905000	-1.560929000
C	-1.094205000	4.004397000	-1.542245000
C	0.454601000	1.892044000	-1.420391000
C	0.050257000	0.501391000	-1.327733000
H	-3.999833000	1.360487000	-1.395558000
H	-4.491518000	3.786277000	-1.520486000
H	-2.678384000	5.455188000	-1.613324000
H	-0.287400000	4.730082000	-1.580386000
C	0.936537000	-0.648303000	-1.225911000
C	1.559095000	-1.378683000	-2.314897000
C	2.433647000	-2.361959000	-1.646907000
C	2.303954000	-2.171096000	-0.264727000
C	3.284605000	-3.344751000	-2.141901000
C	3.016140000	-2.926206000	0.661225000
C	3.996458000	-4.119362000	-1.230595000
H	3.385109000	-3.493967000	-3.212463000
C	3.862723000	-3.906798000	0.149433000

H	2.940247000	-2.743261000	1.727303000
H	4.668868000	-4.892317000	-1.587258000
H	4.437971000	-4.516001000	0.838983000
O	1.616099000	2.325381000	-1.451848000
O	1.376324000	-1.196506000	-3.527001000
N	1.376601000	-1.142704000	-0.038138000
N	-1.305543000	0.420555000	-1.367269000
C	0.973579000	-0.671515000	1.276489000
H	-0.011959000	-0.209606000	1.200888000
H	0.926927000	-1.526654000	1.954249000
C	-2.094502000	-0.780260000	-1.261838000
H	-1.466242000	-1.645820000	-1.490802000
H	-2.908327000	-0.761104000	-1.991725000
C	2.010685000	0.321939000	1.797556000
O	3.068262000	-0.032797000	2.266481000
O	1.593385000	1.565076000	1.617107000
C	-2.667866000	-0.940807000	0.145775000
O	-2.332661000	-0.249749000	1.080201000
O	-3.543680000	-1.930698000	0.157139000
C	2.442925000	2.714404000	1.959374000
C	2.681207000	2.734276000	3.464971000
C	3.737994000	2.657136000	1.156804000
C	1.594597000	3.901734000	1.522621000
H	1.726430000	2.724758000	3.999501000
H	3.280369000	1.879801000	3.783340000
H	3.213822000	3.652785000	3.728749000
H	3.501401000	2.517113000	0.098362000
H	4.269026000	3.606484000	1.276089000
H	4.385589000	1.848508000	1.498072000
H	2.111257000	4.832716000	1.772372000

H	1.430713000	3.862698000	0.442153000
H	0.627547000	3.889717000	2.034226000
C	-4.222458000	-2.335966000	1.398018000
C	-3.190311000	-2.807343000	2.416174000
C	-5.072730000	-1.181264000	1.915219000
C	-5.099569000	-3.492716000	0.938547000
H	-2.554388000	-3.582007000	1.977089000
H	-2.566025000	-1.982749000	2.763350000
H	-3.711973000	-3.237908000	3.275843000
H	-5.740496000	-0.822805000	1.125938000
H	-5.685693000	-1.537926000	2.748133000
H	-4.453088000	-0.354677000	2.265282000
H	-5.663759000	-3.885464000	1.788482000
H	-5.805758000	-3.156801000	0.174124000
H	-4.485708000	-4.296880000	0.523380000

3-GS-E M06-2X/6-31G(d) in acetonitrile

66

-1645.28858951

C	-0.159609000	2.967085000	-0.274427000
C	1.174142000	2.672644000	0.030609000
C	2.142394000	3.673259000	0.057406000
C	1.723273000	4.970473000	-0.230217000
C	0.388876000	5.274567000	-0.539381000
C	-0.567337000	4.266283000	-0.562901000
C	-0.923169000	1.719278000	-0.179905000
C	0.101637000	0.673880000	0.184543000
H	3.183257000	3.451625000	0.265526000
H	2.458305000	5.769337000	-0.222022000
H	0.109940000	6.298009000	-0.764005000

H	-1.607969000	4.472032000	-0.794654000
C	-0.101640000	-0.673871000	0.184535000
C	0.923167000	-1.719262000	-0.179931000
C	0.159611000	-2.967070000	-0.274463000
C	-1.174138000	-2.672637000	0.030586000
C	0.567342000	-4.266264000	-0.562956000
C	-2.142387000	-3.673256000	0.057376000
C	-0.388867000	-5.274551000	-0.539442000
H	1.607973000	-4.472005000	-0.794719000
C	-1.723264000	-4.970465000	-0.230265000
H	-3.183250000	-3.451628000	0.265504000
H	-0.109929000	-6.297990000	-0.764079000
H	-2.458292000	-5.769332000	-0.222075000
O	-2.103457000	1.530156000	-0.427119000
O	2.103451000	-1.530130000	-0.427160000
N	-1.332260000	-1.311369000	0.295260000
N	1.332260000	1.311372000	0.295269000
C	-2.461634000	-0.820938000	1.050107000
H	-2.760118000	-1.581296000	1.784145000
H	-2.179355000	0.076175000	1.600345000
C	2.461631000	0.820931000	1.050115000
H	2.179346000	-0.076180000	1.600351000
H	2.760121000	1.581286000	1.784153000
C	-3.665475000	-0.529469000	0.166205000
O	-3.820488000	-1.013163000	-0.930324000
O	-4.520731000	0.249371000	0.820023000
C	3.665469000	0.529452000	0.166212000
O	3.820486000	1.013146000	-0.930316000
O	4.520726000	-0.249384000	0.820035000
C	-5.773952000	0.683296000	0.201183000

C	-5.471280000	1.497274000	-1.052658000
C	-6.662198000	-0.523290000	-0.084009000
C	-6.395894000	1.565068000	1.277056000
H	-4.750954000	2.286337000	-0.819106000
H	-5.060422000	0.868161000	-1.843344000
H	-6.396289000	1.958546000	-1.411654000
H	-6.795422000	-1.115898000	0.826410000
H	-7.645570000	-0.173605000	-0.412547000
H	-6.235077000	-1.154595000	-0.863918000
H	-7.355987000	1.954280000	0.927607000
H	-6.563229000	0.990454000	2.192644000
H	-5.738024000	2.408305000	1.506098000
C	5.773950000	-0.683308000	0.201201000
C	5.471283000	-1.497265000	-1.052654000
C	6.662210000	0.523274000	-0.083965000
C	6.395875000	-1.565100000	1.277068000
H	4.750949000	-2.286327000	-0.819119000
H	5.060433000	-0.868138000	-1.843333000
H	6.396291000	-1.958538000	-1.411650000
H	6.795429000	1.115869000	0.826463000
H	7.645582000	0.173585000	-0.412497000
H	6.235103000	1.154593000	-0.863871000
H	7.355968000	-1.954315000	0.927622000
H	6.563207000	-0.990500000	2.192665000
H	5.737996000	-2.408334000	1.506092000

3-ES-E wB97XD/6-31G(d) ES optimized in acetonitrile

66

symmetry c1

C	2.349001000	-2.219873000	-1.749646000

C	2.834067000	-1.738947000	-0.524227000
C	4.194085000	-1.576232000	-0.286682000
C	5.066076000	-1.930700000	-1.314955000
C	4.595764000	-2.430025000	-2.536889000
C	3.229536000	-2.580359000	-2.763420000
C	0.886891000	-2.257993000	-1.673263000
C	0.576467000	-1.756664000	-0.320247000
H	4.577102000	-1.197125000	0.654559000
H	6.134193000	-1.816553000	-1.160887000
H	5.304809000	-2.692980000	-3.314996000
H	2.852668000	-2.956530000	-3.709320000
C	-0.683269000	-1.738450000	0.306264000
C	-1.004512000	-2.229516000	1.660704000
C	-2.463241000	-2.133514000	1.746519000
C	-2.936787000	-1.632737000	0.524372000
C	-3.350554000	-2.459635000	2.765996000
C	-4.290967000	-1.416701000	0.296077000
C	-4.711177000	-2.254575000	2.549006000
H	-2.982486000	-2.851271000	3.709075000
C	-5.169384000	-1.736344000	1.330459000
H	-4.665883000	-1.023588000	-0.642658000
H	-5.424800000	-2.489362000	3.331921000
H	-6.233100000	-1.579674000	1.183520000
O	0.075685000	-2.640517000	-2.526257000
O	-0.203092000	-2.645001000	2.507466000
N	-1.855611000	-1.422322000	-0.334462000
N	1.756918000	-1.484848000	0.327325000
C	-1.957143000	-0.584266000	-1.504174000
H	-1.135775000	-0.803180000	-2.186085000
H	-2.878794000	-0.818485000	-2.041302000

C	1.886525000	-0.651851000	1.498015000
H	2.773986000	-0.950345000	2.060952000
H	1.034088000	-0.807409000	2.157943000
C	-1.951066000	0.898549000	-1.131995000
O	-1.808286000	1.309999000	-0.003488000
O	-2.123780000	1.610425000	-2.235069000
C	2.001386000	0.826535000	1.125297000
O	1.965313000	1.241390000	-0.010224000
O	2.147867000	1.530425000	2.237402000
C	-2.201334000	3.079749000	-2.219700000
C	-3.426454000	3.512307000	-1.419345000
C	-0.906913000	3.663667000	-1.663111000
C	-2.364063000	3.422129000	-3.696367000
H	-4.327540000	3.031379000	-1.812722000
H	-3.319053000	3.260137000	-0.362665000
H	-3.548804000	4.596228000	-1.507998000
H	-0.041716000	3.245806000	-2.187692000
H	-0.909235000	4.747913000	-1.811620000
H	-0.810298000	3.456752000	-0.597139000
H	-2.447912000	4.506226000	-3.815797000
H	-1.499636000	3.074492000	-4.270066000
H	-3.266678000	2.956389000	-4.103223000
C	2.358249000	2.986713000	2.226072000
C	3.688771000	3.293819000	1.545230000
C	1.184008000	3.685226000	1.547396000
C	2.409984000	3.328460000	3.710960000
H	4.501923000	2.746648000	2.032358000
H	3.661973000	3.026793000	0.486849000
H	3.896717000	4.365081000	1.628028000
H	0.235654000	3.334354000	1.966880000

H	1.259616000	4.762978000	1.721925000
H	1.186096000	3.503923000	0.472371000
H	2.589474000	4.400202000	3.836160000
H	1.463382000	3.074926000	4.197774000
H	3.217544000	2.780077000	4.205111000

3-MECI SF-PBE0/6-31G(d) in gas phase

66

symmetry c1

C	-2.800287156	1.689842070	0.943043979
C	-1.796064657	2.615398085	0.596881476
C	-2.106812365	3.944469738	0.297004453
C	-3.443913199	4.319794018	0.347342133
C	-4.453576557	3.404295399	0.684330539
C	-4.133974579	2.085623363	0.983434891
C	-2.181884297	0.382255247	1.224684661
C	-0.796799355	0.642602529	1.029144711
H	-1.343697256	4.666357680	0.017748904
H	-3.711241767	5.347418057	0.114996720
H	-5.488378008	3.734552023	0.708899823
H	-4.896437642	1.360041352	1.253749058
C	0.256337561	-0.334608748	1.105075900
C	1.076224097	-0.706453986	2.322386864
C	1.964966527	-1.790697417	1.872850447
C	1.683179065	-2.017590820	0.524431301
C	2.935350588	-2.531955571	2.525697937
C	2.333563413	-2.984770193	-0.218252656
C	3.618631396	-3.505996888	1.793350283
H	3.148655997	-2.350950371	3.574889493
C	3.316156123	-3.726493641	0.449365896

H	2.065432054	-3.196348230	-1.246634382
H	4.386373611	-4.103552715	2.275191225
H	3.848188061	-4.499033370	-0.097771992
O	-2.763639518	-0.687227287	1.520544072
O	1.009274144	-0.149138851	3.398798834
N	0.663132154	-1.106485437	0.110192764
N	-0.576981962	1.973849832	0.628270421
C	0.140109190	-1.052776252	-1.248613287
H	-0.355323084	-0.091812507	-1.372259090
H	0.985108991	-1.128575860	-1.936685085
C	0.702590860	2.604325657	0.636848103
H	1.268399491	2.339417729	1.539769043
H	0.572688704	3.691722152	0.664821502
C	-0.818316448	-2.228201478	-1.477237856
O	-0.416847736	-3.360676724	-1.637539549
O	-2.057381538	-1.796615290	-1.449813819
C	1.544990885	2.276063357	-0.594514195
O	1.096522086	1.848804198	-1.633796028
O	2.826363438	2.539623423	-0.338159568
C	-3.209360148	-2.721183047	-1.400468195
C	-3.289946203	-3.491223772	-2.710714555
C	-3.064908489	-3.619327228	-0.179621377
C	-4.381761373	-1.769430071	-1.225987158
H	-3.338285971	-2.800468551	-3.559865365
H	-2.432529219	-4.156069039	-2.837191888
H	-4.203706416	-4.095284962	-2.715406429
H	-2.908481820	-2.994500526	0.706140248
H	-3.990148321	-4.190495181	-0.047247322
H	-2.237903351	-4.324025020	-0.294276631
H	-5.314313296	-2.341792757	-1.180567917

H	-4.255957476	-1.206047624	-0.296120478
H	-4.445158787	-1.070960196	-2.067412124
C	3.863338143	2.356836974	-1.356435056
C	3.884802802	0.911683396	-1.846642942
C	3.635842214	3.345481679	-2.493767340
C	5.142087809	2.679538528	-0.596195553
H	3.934252943	0.219449169	-0.998475085
H	3.001857014	0.682371136	-2.445919094
H	4.776308761	0.753814379	-2.463245597
H	3.614776804	4.370755670	-2.109131615
H	4.455929798	3.269096876	-3.215636755
H	2.696269800	3.139390763	-3.011302151
H	6.006996647	2.586795562	-1.260511096
H	5.111928681	3.700651860	-0.203211312
H	5.274763157	1.990449696	0.244574850
